# Nuclear and chloroplast DNA phylogeography suggests an Early Miocene southward expansion of *Lithocarpus* (Fagaceae) on the Asian continent and islands

**DOI:** 10.1186/s40529-018-0244-8

**Published:** 2018-11-08

**Authors:** Chih-Kai Yang, Yu-Chung Chiang, Bing-Hong Huang, Li-Ping Ju, Pei-Chun Liao

**Affiliations:** 1 Department of Life Science, National Taiwan Normal University, 88, Ting-Chow Rd, Sec 4, Taipei, 116 Taiwan; 2 The Experimental Forest, College of Bio-Resources and Agriculture, National Taiwan University, 12, Sec. 1, Chien-Shan Rd., Nantou, 55750 Taiwan; 3 Department of Biological Sciences, National Sun Yat-sen University, 70, Lien-Hai Rd., Kaohsiung, 80424 Taiwan; 4 Botanical Garden Division, Taiwan Forestry Research Institute, 53, Nan-Hai Rd., Taipei, 10066 Taiwan

**Keywords:** Stone oak, Continental Asia, Dispersal–extinction–cladogenesis (DEC), Diversification rate, Endemism, Greater Sunda Islands, Historical biogeography, Indochina, *Lithocarpus*, Phylogeny

## Abstract

**Background:**

Most genera of Fagaceae are thought to have originated in the temperate regions except for the genus *Lithocarpus*, the stone oaks. *Lithocarpus* is distributed in subtropical and tropical Asia, and its ancestral population is hypothesized to be distributed in tropical regions in Borneo and Indochina. Borneo and the nearby islands (the Greater Sunda Islands) were connected to the Malay Peninsula and Indochina prior to the Pliocene epoch and formed the former Sundaland continent. The Southeast Asian *Lithocarpus*, is thought to have dispersed between continental Asia and the present Sundaland. The drastic climate changes during the Pliocene and Pleistocene epochs which caused periodic sea-level changes is often used to explain the cause of its diversity. The aim of this study was to establish phylogenetic relationships by analyzing nuclear (nrDNA) and chloroplast (cpDNA) DNA in order to describe and analyze the origin, causes of diversification and historical biogeography of *Lithocarpus.*

**Results:**

Phylogeny reconstructed through the multiple-species coalescent method with nrDNA and cpDNA revealed that the continental-Asian taxa were clustered at the basal lineages. The derived lineages of tropical *Lithocarpus*, with the inference of a subtropical ancestral state, imply a southward migration in the Early Miocene period with subsequent in situ diversification in the Greater Sunda Islands. The gradual decrease in temperature since the Middle Miocene period is proposed as a cause of the increase in the net diversification rate.

**Conclusions:**

The historical ancestral origin of *Lithocarpus* has been suggested to be mainland Asia. Southward migration in the Early Miocene period with subsequent in situ diversification could explain the current diversity of stone oaks in Southeast Asia. This study also considered the multiple origins of stone oaks currently indigenous to the subtropical islands offshore and near mainland China. Our results provide phylogenetic evidence for a subtropical origin of Asian stone oaks and reveal the process of diversification and how it fits into the timeline of major geologic and climatic events rather than local, episodic, rate-shifting events.

**Electronic supplementary material:**

The online version of this article (10.1186/s40529-018-0244-8) contains supplementary material, which is available to authorized users.

## Background

Dispersal and vicariance are the main biogeographic factors that contribute to species distribution (Donoghue et al. 2001; Manos and Stanford 2001). Quaternary glacial cycles that caused topographic changes and climatic fluctuations should have affected the distribution of species by both dispersal and vicariance (Hewitt 2004). For example, the well-known retreat of European white oaks into the southern refugia during glacial periods and their interglacial northward expansion explains the reshuffling of species distribution (Petit et al. 2002a, b). In Southeast Asia, the emergence of ancient Sundaland, the former continent connecting the Thai-Malay peninsula to Borneo and the Philippine archipelagos, was suggested as a land bridge for species dispersal between Indochina and the Malay Archipelago, but the submergence of the Sunda shelf was the prime barrier for vicariant events between the Malay Archipelago and continental Asia (Lohman et al. 2011). Glacially-dependent demographic changes leading to genetic differentiation of populations were also reported for several eastern and Southeast Asian land plants, such as *Ceriops tagal* (Rhizophoraceae) (Liao et al. 2011), *Castanopsis carlesii* (Fagaceae) (Cheng et al. 2005), *Dysosma versipellis* (Berberidaceae) (Fu et al. 2009), *Machilus* spp. (Lauraceae) (Wu et al. 2006), and *Pinus massoniana* (Pinaceae) (Ge et al. 2012).

In addition to the topographic changes in Southeast Asia (e.g., Sundaland), past climate changes also affected the flora of subtropical China and adjacent areas (Qiu et al. 2011). In their report, Qiu et al. (2011) summarized the evidence that Southwest China, which is adjacent to the Indochinese Peninsula, has the greatest floral biodiversity. The complex geohistory and physiographical heterogeneity of Southwest China provided multiple refuges for plant populations (e.g., Nanling Mts., Yungui Plateau, Hengduan Mts., etc.) during Quaternary glacial periods in terms of inter- and postglacial northward, eastward, and westward recolonization (Qiu et al. 2011). Past climate changes also severely affected the biogeographic patterns of Southeast Chinese, Japanese, Korean, and Taiwanese plants (reviewed by Chiang and Schaal 2006; Qiu et al. 2011) such as *Cunninghamia konishii* (Cupressaceae) (Chung et al. 2004), *Ainsliaea* spp. (Asteraceae) (Mitsui and Setoguchi 2012), *Picea jezoensis* (Pinaceae) (Aizawa et al. 2007), *Pinus luchuensis* complex (Pinaceae) (Chiang et al. 2006), and *Ligularia hodgsonii* (Asteraceae) (Wang et al. 2013). The distribution of these plants was probably due to dispersal/vicariance events driven by the emergence/submergence of the last glacial East China Sea land bridges (Qiu et al. 2011); similar events occurred among fauna (Millien-Parra and Jaeger 1999).

Stone oaks (*Lithocarpus* Bl., Fagaceae) are the most widely distributed trees in East and Southeast Asia ranging from northeast India to South China, southern Japan and Taiwan, Indochina, Borneo, and Papua New Guinea (Nixon 1989). Across this range, severe topography changes took place during the Quaternary glacial/interglacial cycles. In older studies, *Lithocarpus* was supposed to have had a tropical origin in Southeast Asia and an Asian migration which occurred at least twice: a westward dispersal to Europe identified based on the fossil records (no extant species) and an eastward colonization of western North America (one extant species) (Cannon and Manos 2003). However, the American species has been identified as a novel genus *Notholithocarpus* because of its distant phylogenetic relationship to the Asian species (Manos et al. 2008); therefore, the rest of the Asian *Lithocarpus* species formed a monophyly (Manos et al. 2001, 2008; Oh and Manos 2008). *Lithocarpus* sensu lato is composed of approximately 300 species and is the second largest genus of Fagaceae (Nixon 1989).

Manos and Stanford (2001) claimed that the most ancient haplotypes were distributed in northern Borneo and Indochina, which then separated into the Borneo population and the Indochina population in the middle Miocene, during which Sundaland was narrowed and the Gulf of Thailand appeared. After vicariance, the prolonged in situ diversification increased the frequency of endemics (Cannon and Manos 2003). Cannon and Manos (2003) also proposed a recent diversification of the genus *Lithocarpus* according to unresolved gene trees of cpDNA (chloroplast DNA) and nrITS (nuclear ribosomal internally transcribed spacers). However, their inference was mainly based on a study using very biased sampling in Borneo with only a few samples from continental Asia. According to several case studies regarding the isolation and migration of plant species between continental Asia and the Greater Sunda Islands, the diversification of Southeast Asian *Lithocarpus* might be related to recurrent separation and dispersal between geographical-transitional species congeners. Such recurrent secondary contacts were also highlighted for insular species diversity (e.g., the origin of Taiwanese endemic species, Chiang et al. 2012; Chung et al. 2007). The relatively high diversity and endemism of *Lithocarpus* in continental islands near mainland China provide opportunities to test the hypotheses of the origin of insular forest species, either multiple origins by continent-island colonization or single origins with in situ radiation.

In this study, we used East and Southeast Asian stone oaks (*Lithocarpus*) as subjects to determine whether phylodiversity was related to biogeographic events regarding climatic-related geographic timelines. Phylogenetic analyses of *Lithocarpus* (Fagaceae) based on maternally inherited plastid markers and biparentally inherited nuclear markers were assessed to explore the biogeographic patterns via phylogeographical analyses. Based on surveys of topological and temporal analyses, we proposed answers to the following questions: (1) Are there any diversification rate shifts in *Lithocarpus* in different geographical regions? (2) Do the rate-shift events correspond to any geographical event? (3) How is the insular diversity generated?

## Methods

### Plant material sampling and molecular techniques

Leaf samples were collected from stone oak populations covering the areas of Nanling (E113°01′15′′, N24°58′51′′), Mangdang Shan (E118°07′33′′, N26°41′40′′), Emei (E103°16′27′′, N29°34′52′′), Chinfo Shan (E107°09′43′′, N29°01′04′′), Kunming Golden Temple (E102°46′10′′, N25°05′15′′), and botanical gardens (Additional file 1: Table S1). Total genomic DNA was extracted from leaf samples using a modified cetyl trimethylammonium bromide (CTAB) method (Doyle and Doyle 1987). The DNA samples were amplified by polymerase chain reaction (PCR) using primers specific for the regions of the *atp*B-*rbc*L spacer of the chloroplast (cp) genome and the nuclear ribosomal internal transcribed spacer (nrITS) of the nuclear genome. PCR products of the *atp*B-*rbc*L spacer were directly sequenced. The nrITS amplicons were cloned using the yT&A cloning kit (Yeastern Biotech, Taipei, Taiwan) and sequenced with M13F and M13R primers. Sequencing reactions were performed using the ABI BigDye 3.1 Terminator Cycle Sequencing Kit (Applied Biosystems, Foster City, CA, USA) and the fragments were sequenced from both directions with the ABI PRISM^®^3730XL DNA Sequencer (Perkin-Elmer, Foster City, CA, USA). All sequence polymorphisms were visually rechecked from the chromatograms.

### Sequence alignment

Chromatograms of DNA sequences were inspected by SeqMan implemented in DNASTAR ver. 7.0 (Lasergene, Germany). Genetic confirmation of each sequence was done by querying the nucleotide database in NCBI using the BLASTN program and NetPlantGene server in the Center for Biological Sequence Analysis (http://www.cbs.dtu.dk/services/NetPGene/). All sequences were deposited in the NCBI nucleotide sequence database under the following accession numbers: KF992718–KF992796 (*atp*B-*rbc*L) and KJ685163–KJ685212 (nrITS). To enrich the sampling number, 108 sequences of *atp*B-*rbc*L and 77 sequences of nrITS from NCBI were incorporated for analyses. Sequence alignments were performed by Clustal X (Thompson et al. 1997) and manually edited using the BioEdit ver.7.0.9.0 (Hall 1999). Long fragments of sequences were trimmed to equal alignment lengths for subsequent phylogenetic analyses.

### Phylogenetic analyses

As the taxa we collected for chloroplast and nuclear phylogenetic analyses were not exactly identical, multi-species coalescent phylogenetic trees (Drummond and Rambaut 2007; Heled and Drummond 2010) were reconstructed based on combinations of genomic markers and the two target markers separately. Consequently, three trees were developed as data structures for further analysis: the cpDNA + nrITS tree (64 species), the cpDNA tree (81 species), and the nrITS tree (70 species). Since extinction events in *Lithocarpus* are rare (Cannon and Manos 2003; Xing et al. 2014), it is likely that the rare event is overestimated from incomplete taxon sampling. Therefore the extinctions were deliberately excluded from species tree reconstruction to explain the species diversification, and we considered only cladogenesis and biogeographical factors. Yule’s pure-birth model that ignores the death (extinction) rate parameter was adopted for phylogeny reconstruction. The general-time-reversible substitution model (GTR) together with a gamma and invariant site heterogeneity model by BEAST ver. 1.7.3 (Drummond and Rambaut 2007) were used. Since the ucld.stdev parameter estimate deviated from zero, to account for the rate of heterogeneity among lineages in both markers (95% HPD of ucld.stdev = 0.849 ~ 1.289 and 0.759 ~ 1.407 in ITS and cpDNA, respectively) we have used the uncorrelated lognormal (UCLN) relaxed clock model instead of the strict clock model to estimate divergence times of about 40 Mya from the outgroup genus *Chrysolepis* (Kvaček and Walther 1989). Five independent pre-runs of ten-million generations of the length of MCMC were performed to obtain better parameter priors for the following three independent 50-million generations of the MCMC process. Genealogies for each lineage were sampled every 10,000 generations and the initial 10–25% of the sample run was discarded as burn-in according to the simulation trajectory. The output data containing all the statistics were summarized by TRACER ver. 1.5 (Rambaut and Drummond 2003) and both log and tree files of the last five runs were combined using LogCombiner ver. 1.6.1 (Drummond and Rambaut 2007). We resampled the originally simulated trees with a sampling frequency of 8000 and 6000 trees for ITS and cpDNA, respectively, to reduce the number of trees by LogCombiner ver. 1.6.1 and the posterior samples of trees were summarized by TreeAnnotator ver. 1.6.1 (Drummond and Rambaut 2007). FigTree ver. 1.3.1 (Rambaut 2008) was used to display the summarized trees.

### Biogeographic inferences

We applied two strategies to infer the biogeographic events of Asian stone oaks: the statistical dispersal-vicariance (S-DIVA) model and the dispersal–extinction–cladogenesis (DEC) model, performed by RASP (Yu et al. 2015) and LAGRANGE (Matzke 2014) software, respectively. As described above, the extinction rate has probably been misidentified and overestimated by incomplete taxon coverage. However, Yule’s pure-birth model is a better fit to our data than the birth-and-death process as suggested by ∆AIC_RC_ test statistics (Table 2). Therefore ‘extinction’ was not considered in this study when reconstructing the phylogenetic tree for subsequent DEC analysis.

**Fig. 1:**
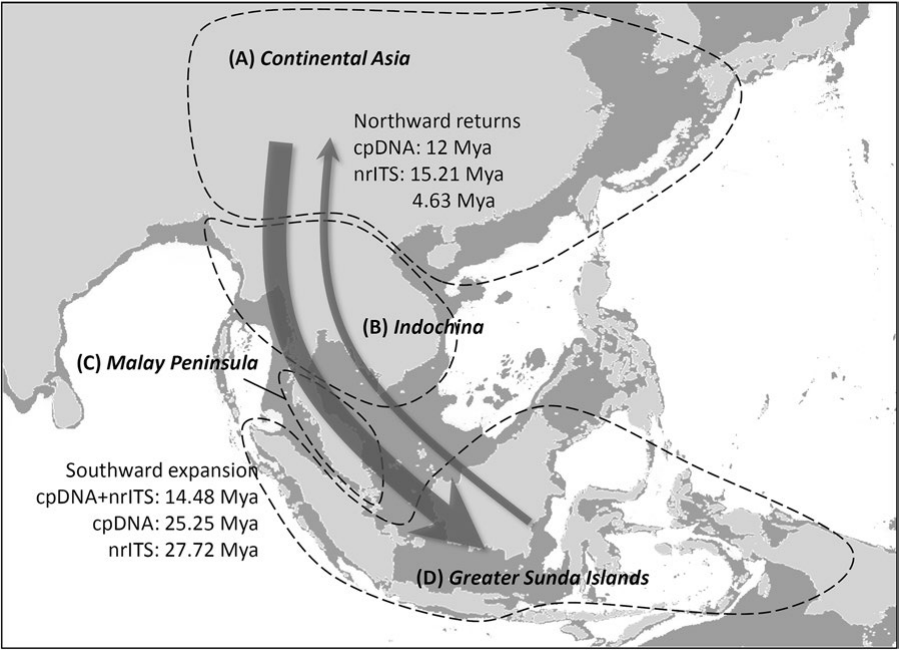
Geographic distribution and the probable directions of species dispersal. Geographic distribution of the Asian *Lithocarpus* is separated into **A** continental Asia, **B** Indochina, **C** the Malay Peninsula, and **D** the Greater Sunda Islands. Arrows point to the putative dispersal routes and the migration times estimated by cpDNA and nrITS are denoted. Light gray regions are the present landmass; dark gray regions are the shallow seabed, representing the hypothetical coastlines of continental Asia and Sundaland during the Miocene and Quaternary glacials

Ancestral area reconstruction was performed using both S-DIVA and DEC models. The study species were mapped into four ancestral areas: (A) continental Asia (including mainland China, northern India, and the islands off continental Asia), (B) Indochina, (C) the Malay Peninsula, and (D) the Greater Sunda Islands (including Borneo, the Philippines, Sumatra, Java, and other Indonesian archipelagos) (Fig. 1). The Greater Sunda Islands and Indochina were Cannon and Manos’s (2003) hypothetical ancestral areas; extensions of the Malay Peninsula connected to the Greater Sunda Islands and Indochina form ancient Sundaland. Continental Asia is the major section where most of the sympatric speciation occurs in *Lithocarpus* with other species of Fagaceae, and it is an alternative ancestral area for *Lithocarpus*.

### Homogeneity tests for whole-tree diversification rates

The asymmetric rate shift of nodes of the species tree was estimated using the taxon-size insensitive (TSI) model and equal-rate Markov (ERM) random branching model of the SymmeTree program (Moore and Chan 2005). The Colless’s tree imbalance index (*I*_C_), the nodal probability product (*M*_Π_ and the modified version *M*_Π_*), and sum (*M*_Σ_ and *M*_Σ_*) were used to display the diversification rate variation of the whole tree (Chan and Moore 2002; Moore et al. 2004). Homogeneous evolutionary rates of descendant clades from the common ancestor (the node) were tested with delta-shift statistics (Δ1 and Δ2) at all nodes (Moore et al. 2004). One million random resolutions with 1000 TSI/ERM-resolved trees under one-million ERM simulations were performed to identify significant diversification rate shifts of each node. We selected the best diversification rate model (rate-constant, RC, or rate-variable, RV) through ∆AIC_RC_ test statistics using the *fitdAICrc* function of R package laser ver. 2.3 (Rabosky 2006). The *fitdAICrc* tested the Yule’s pure-birth (pureBirth) and birth–death (bd) models of RC, and the exponential and logistic variants of the density-dependent speciation rate models (DDX and DDL) and multi-rate variants of the pure-birth model (yule2rate and yule3rate) of RV.

### Temporal-based diversification-rate-shift analyses

A temporal analysis that accumulates speciation events (lineages) through time was performed to infer the emergence time of species using lineage-through-time (LTT) analysis. The LTT analysis was performed in R using ape package (Paradis et al. 2004). We also resampled 1000 trees from the simulated-trees file by LogCombiner to display the LTT distribution of simulated results using the function *mltt.plot* in ape. Chronograms of the species tree reconstructed by BEAST were used as input trees. Effective sizes of lineages over time were also estimated using the reversible-jump Markov chain Monte Carlo computation (rjMCMC) (Opgen-Rhein et al. 2005) by the function *mcmc.popsize* of ape. One-million-step Markov-chain simulations were performed for the rjMCMC estimation, and the first 1000 steps were dropped as burn-in, with a setting thinning factor = 1000, for both whole tree topologies and subclades.

### Diversification rate estimation by Bayesian analysis of macroevolutionary mixtures (BAMM)

Since the number of species used for reconstructing the species tree (63 species) is roughly 20% of the total number of *Lithocarpus* species recognized in the world (~ 300 species), we recalculated the net diversification rate with 20% sampling fractions by the BAMM approach (Rabosky et al. 2014). For estimating the net diversification rate, 5,000,000 Markov chain Monte Carlo simulations with 1/1000 thinning and 10% burn-in were set. The phylorate plot that shows the speciation rate along branches of the phylogeny was drawn with the Jenks natural breaks method. A histogram of rate heterogeneity was also drawn to illustrate proportions of lineages of rate clusters in the phylogenetic tree. All of the diversification rate analyses were conducted with the assistance of the BAMM program and R package BAMMtools (Rabosky et al. 2014).

## Results

### Phylogenetic inferences

A total of 187 sequences of cp *atp*B-*rbc*L fragment from 81 species and 132 sequences of nrITS from 70 species were determined for phylogenetic, biogeographic, and diversification rate shift analyses. The tree topologies inferred from individual markers were significantly different (Additional file 1: Figure S1), and a strong heterogeneity and incomplete lineage sorting was found under different evolutionary histories (Hoelzer 1997; Page and Charleston 1998). Therefore, divergence time estimates for biogeographic events were calculated with a strategy that analyzed both markers via the multiple-species coalescent analysis implemented in *BEAST.

**Fig. 2:**
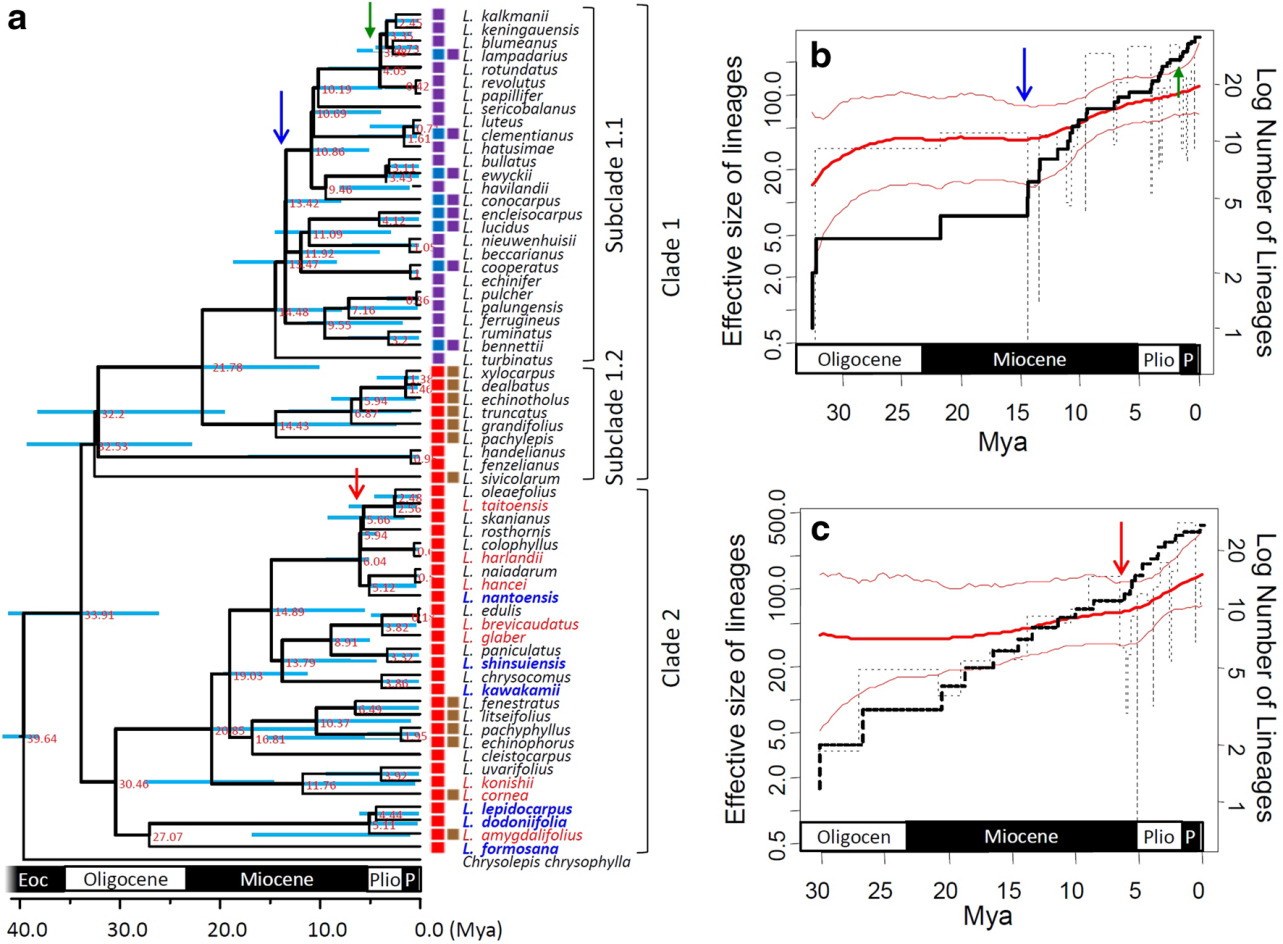
Species tree and temporal analysis of diversification rates inferred by both cpDNA *atp*B-*rbc*L and nrITS. **a** Species tree reconstructed under the Yule’s pure-birth speciation model. Bold lines indicate lineages grouping with posterior probability > 80%; node labels are the splitting time (mya); the node bar is the 95% highest posterior density interval (HPD) of the splitting time. Geographic distribution areas are displayed as colored boxes. **b** and **c** are skylines of diversification rates of clade 1 and clade 2, respectively, inferred by rjMCMC and LTT. Dashed lines represent the classic skyline plots, while the bold and thin red lines indicate the skylines estimated with the rjMCMC and the corresponding 95% confidence intervals, respectively. The bold black line is the LTT plot. The blue, green and red arrows indicate the phylogenetic locations and times of diversification rate shifts

**Fig. 3:**
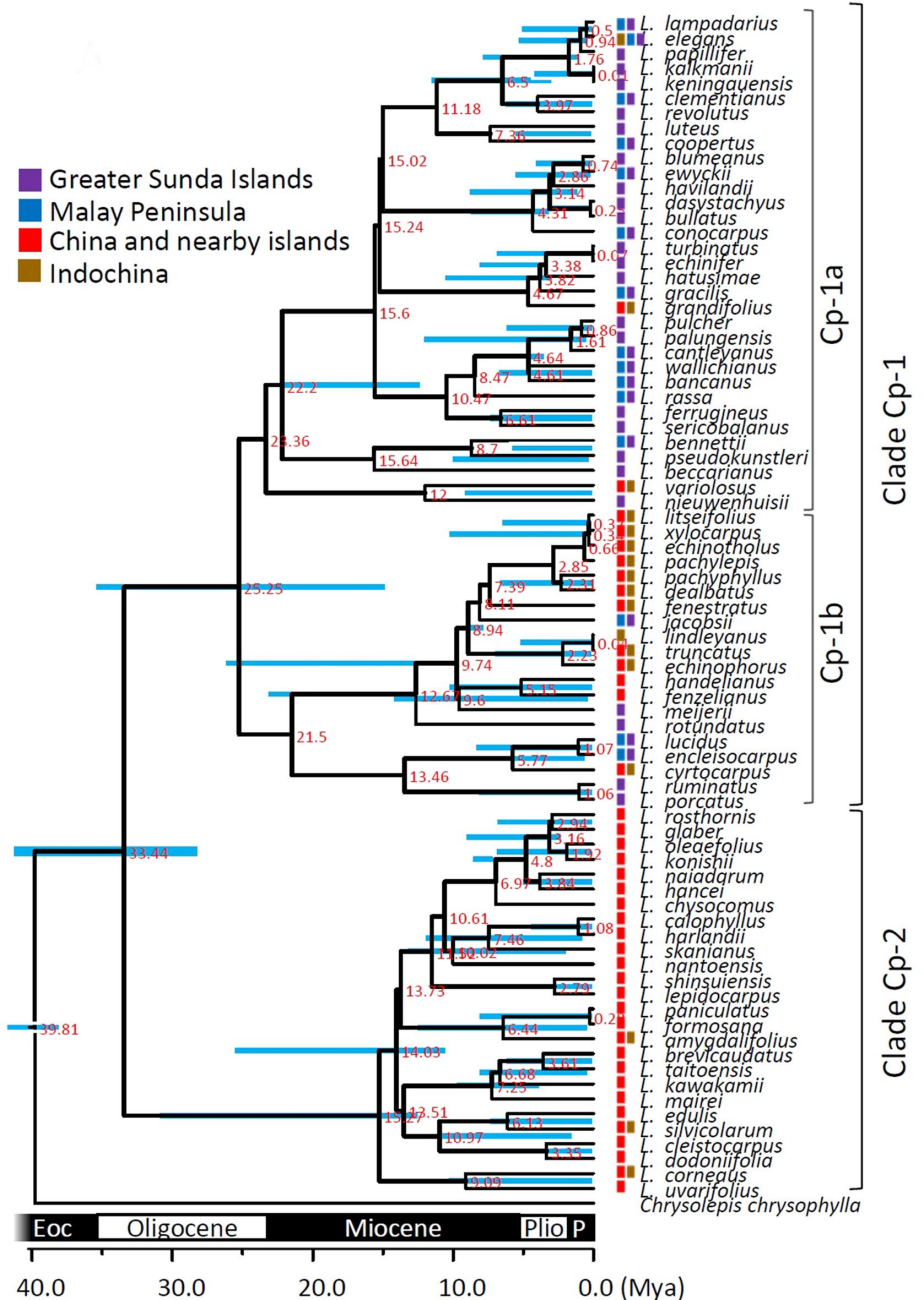
Species tree and temporal analysis of diversification rates inferred by cpDNA *atp*B-*rbc*L spacer reconstructed under Yule’s pure-birth speciation model. Bold lines indicate the lineages grouping with posterior probability > 80%; node labels are the splitting time (unit: mya); node bar is the 95% highest posterior density interval (HPD) of the splitting time. Geographic distribution areas were displayed as the colored box

**Fig. 4:**
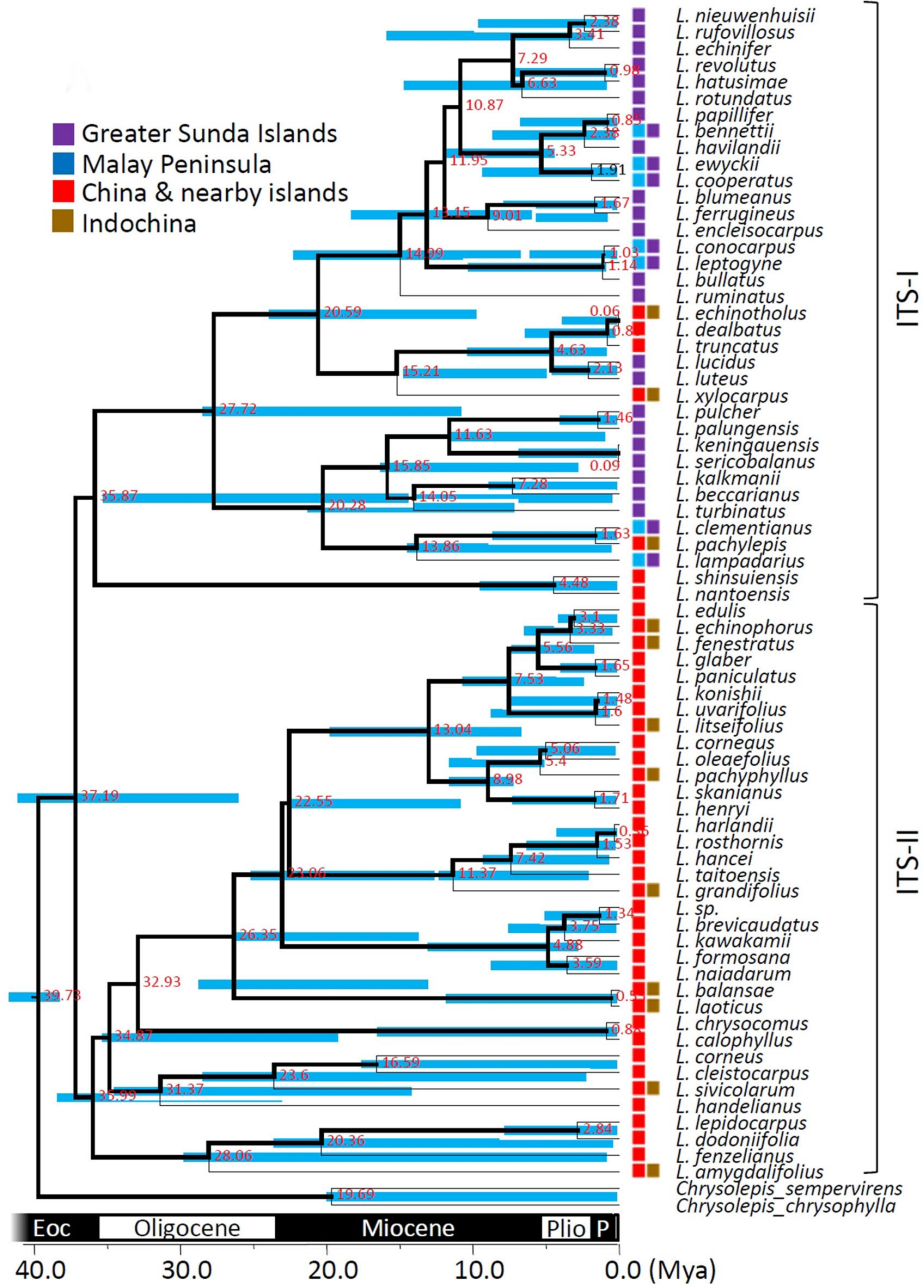
Species tree and temporal analysis of diversification rates inferred by nuclear ITS spacer reconstructed under the Yule’s pure-birth speciation model. Bold lines indicate the lineages grouping with posterior probability > 80%; node labels are the splitting time (mya); the node bar is the 95% highest posterior density interval (HPD) of the splitting time. Geographic distribution areas are displayed as the colored boxes

The splitting time of *Lithocarpus* estimated by multi-species coalescent methods with a combined cpDNA and nrITS tree (hereafter: combined cpDNA and nrITS data) was about 33.91 Mya [95% highest posterior density interval (95% HPD): 25.98–41.35 Mya], which was probably around the time of the Eocene–Oligocene boundary (known as the Grande Coupure; it is marked by the Eocene–Oligocene extinction event), by rooting with the outgroup *Chrysolepis chrysophylla* at *c.* 40 Mya based on the relaxed-clock model (Fig. 2a). The coalescent estimation is consistent with the estimates formed from individual markers cpDNA (33.44 Mya, 95% HPD: 28.23–41.27 Mya) and nrITS (37.19 Mya (95% HPD: 25.89–41.28 Mya). Since that time, *Lithocarpus* has been divided into two clades; the species that make up the first clade are widespread in continental Asia, the continental islands (Japan, Taiwan, and Hainan), the Southeast Asian islands (including the Greater Sunda Islands and the Philippines), and these are shown as clade 1 (cpDNA + nrITS), clade cp-1 (cpDNA), and clade ITS-I (nrITS) respectively (Figs. 2, 3, 4). The second clade is composed of species that are restricted in their geographic distribution to China and the nearby islands (Japan, Taiwan, and Hainan), and these are displayed as clade 2, clade cp-2, and clade ITS-II (Figs. 2a, 3, and 4) respectively. The splitting times of other species that were isolated from their geographic distribution in the Malay Peninsula, the Greater Sunda islands, and the Philippines grouped in subclade 1.1 might have coalesced back to 14.48 Mya (95% HPD: 7.66–19.22 Mya) (Fig. 2a), roughly at the end of the Middle Miocene disruption (14.8 ~ 14.5 Mya). Within clade cp-1 of the cpDNA tree, subclades cp-1a and cp-1b are evident at 25.25 Mya (95% HPD: 14.77–35.54 Mya), during the Late Oligocene period (Fig. 3). However, no sub-clades can be distinguished from clade ITS-I of the nrITS tree (Fig. 4).

### Whole-tree diversification rate homogeneity tests

Testing the rate of homogeneity of the whole-tree diversification is the first step in evaluating the cladogenesis imbalance of the independent clades (Ricklefs 2007). Two analytical strategies, the topological analysis of the tree imbalance and the ∆AIC_RC_ statistic, were used for the whole-tree rate homogeneity tests. Table 1 summarizes the distribution of ERM simulations for five tree-imbalance indices. Tail probabilities of all indices are < 0.001 in the tree inferred from combined cpDNA and nrITS data sets, indicating the rejection of rate homogeneity of the whole trees (Table 1). However, no significant imbalance in the number of descendants was derived from any node when tested by delta-shift statistics (data not shown), suggesting a gradual change in diversification rates but not a punctuated diversification (Moore et al. 2004).

**Table 1:** Tail probabilities of asymmetric values for the among-lineage diversification rate variation in the phylogenetic topologies inferred from the species tree (BEAST)

	*I*_*C*_	*M*_Π_*	*M*_*Π*_	*M*_Σ_*	*M*_*Σ*_
Both cpDNA and nrITS (Fig. 2a)					
Observed	251	− 0.5855	1.50E−10	0.6747	50.2261
Min ERM	507	− 0.9566	1.93E−17	0.5302	44.0535
Max ERM	64	− 0.0856	0.0472	0.9256	61.3567
0.025 frequentile RR	368	− 0.8315	1.08E−13	0.5918	47.8035
0.975 frequentile RR	233	− 0.5207	4.01E−08	0.7284	54.2252
TailPr	0.0006	0.0003	3.00E−05	0.0001	6.00E−05
cpDNA (Fig. 3)					
Observed	324	− 0.5129	4.33E−10	0.7196	66.2184
Min ERM	651	− 0.9533	5.95E−19	0.5418	55.7200
Max ERM	104	− 0.1478	0.0010	0.8833	73.5864
0.025 frequentile RR	440	− 0.7518	1.98E−15	0.6262	60.3685
0.975 frequentile RR	227	− 0.3930	2.91E−08	0.7900	68.394
TailPr	0.0051	0.0005	8.00E−05	0.0002	0.0002
nrITS (Fig. 4)					
Observed	294	− 0.5483	1.43E−10	0.7035	58.2993
Min ERM	589	− 1.0168	2.51E−19	0.5244	50.1407
Max ERM	79	− 0.1281	0.0020	0.8990	67.3080
0.025 frequentile RR	386	− 0.7540	3.50E−14	0.6198	55.2110
0.975 frequentile RR	215	− 0.4135	9.37E−08	0.7744	62.4962
TailPr	0.0044	0.0023	0.0037	0.0059	0.0048
cpDNA (Additional file 1: Figure S1A)					
Observed	599	− 0.5573	3.72E−19	0.6878	103.0830
Min ERM	1112	− 0.8805	1.77E−28	0.5576	94.1847
Max ERM	220	− 0.2034	2.54E−07	0.8536	117.8260
0.025 frequentile RR	854	− 0.7510	2.40E−23	0.6348	100.578
0.975 frequentile RR	463	− 0.4276	8.03E−14	0.7606	110.5650
TailPr	0.0007	3.00.E− 05	0.0001	0.0007	0.0022
nrITS (Additional file 1: Figure S1B)					
Observed	724	− 1.0221	2.02E−21	0.5400	60.0154
Min ERM	754	− 1.0083	5.14E−20	0.5637	60.8534
Max ERM	120	− 0.1426	0.0004	0.8893	80.0882
0.025 frequentile RR	652	− 0.9238	9.54E−20	0.5688	62.8712
0.975 frequentile RR	351	− 0.5370	1.53E−12	0.6977	70.1858
TailPr	0.0021	0.0005	0.0019	0.0017	0.0144

*I*_*C*_ is the Colless’s tree imbalance index; *M*_*Π*_ and *M*_*Σ*_ are the nodal probability product and nodal probability sum of the tree, respectively; *M*_Π_* and *M*_Σ_* are modified versions of *M*_*Π*_ and *M*_*Σ*_ obtained through differential weighting of the individual equal-rate Markov nodal probabilities according to their species diversity. These indices display the diversification rate variation of the whole tree

The inference of diversification rate heterogeneity of *Lithocarpus* was also supported by positive ∆AIC_RC_ values of the trees assessed from combined marker sets (Table 2). The yule3rate of the RV model had a higher likelihood than other RC and RV models and showed the highest net diversification rate at stage 2 (r2) in all trees (r2 = 9.936 in Fig. 2a and Table 2). A very short time span (stage 1–stage 2 [st1–st2]) of diversification rate increase was estimated in spite of quite different estimated times for each tree (st1 = 5.939 Mya, st2 = 5.936 Mya in Fig. 2a and Table 2).

**Table 2:** Testing for diversification rate variation models by ∆AIC_RC_ test statistic

Model	Log-likelihood	AIC	Parameter
Both cpDNA and nrITS (Fig. 2a)			
PureBirth	− 2.3419	*6.684*	r1 = 0.101
bd	− 1.682121	7.364	r1 = 0.076, a = 0.405
DDX	− 1.972169	7.944	r1 = 0.065, x = − 0.136
DDL	− 2.342	8.684	r1 = 0.101, k = 1,168,951
yule2rate	− 1.083	8.166	r1 = 0.0687, r2 = 0.112, st1 = 14.894
yule3rate	1.871	*6.258*	r1 = 0.087, r2 = 9.936, r3 = 0.115, st1 = 5.939, st2 = 5.936
∆AIC_RC_		0.426	
cpDNA (Fig. 3)			
PureBirth	25.97718	− *49.95435*	r1 = 0.120
bd	26.15159	− 48.30319	r1 = 0.105, a = 0.220
DDX	26.19223	− 48.38446	r1 = 0.091, x = − 0.082
DDL	25.97713	− 47.95426	r1 = 0.120, k = 1,428,290
yule2rate	27.806	− 49.612	r1 = 0.063, r2 = 0.130, st1 = 15.636
yule3rate	31.18926	− *52.37852*	r1 = 0.063, r2 = 0.665, r3 = 0.124, st1 = 15.636, st2 = 15.017
∆AIC_RC_		2.424	
nrITS (Fig. 4)			
PureBirth	− 5.870249	13.7405	r1 = 0.086
bd	− 4.303511	*12.60702*	r1 = 0.058, a = 0.532
DDX	− 5.695618	15.39124	r1 = 0.061, x = − 0.105
DDL	− 5.870443	15.74089	r1 = 0.086, k = 1,284,297
yule2rate	− 2.426486	10.85297	r1 = 0.073, r2 = 0.148, st1 = 2.380
yule3rate	1.189585	*7.62083*	r1 = 0.075, r2 = 0.471, r3 = 0.112, st1 = 1.713, st2 = 1.457
∆AIC_RC_		4.986	
cpDNA (Additional file 1: Figure S1A)			
PureBirth	103.309	− 204.617	r1 = 0.121
bd	107.116	− *210.231*	r1 = 0.076, a = 0.574
DDX	106.169	− 208.338	r1 = 0.046, x = − 0.251
DDL	103.308	− 202.616	r1 = 0.121, k = 2,195,621
yule2rate	108.500	− 211.000	r1 = 0.084, r2 = 0.153, st1 = 7.286
yule3rate	112.757	− *215.514*	r1 = 0.095, r2 = 0.184, r3 = 0.024, st1 = 4.059, st2 = 0.328
∆AIC_RC_		5.283	
nrITS (Additional file 1: Figure S1B)			
PureBirth	27.403	− 52.806	r1 = 0.110
bd	31.284	− *58.568*	r1 = 0.060, a = 0.667
DDX	29.731	− 55.463	r1 = 0.038, x = − 0.306
DDL	21.496	− 38.991	r1 = 0.140, k = 174
yule2rate	31.106	− 56.213	r1 = 0.103, r2 = 0.368, st1 = 0.227
yule3rate	34.049	− *58.096*	r1 = 0.093, r2 = 9.384, r3 = 0.172, st1 = 2.055, st2 = 2.053
∆AIC_RC_		− 0.471	

r1, r2, r3, net diversification rates at stages 1, 2, and 3; st1 and st2, the first and the second rate-shift times; a, the extinction fraction extinction rate/speciation rate; x, the x parameter in the density-dependent exponetial model; k, the K parameter in the logistic density dependent model

*PureBirth* pure birth (Yule) model, *bd* rate-constant birth–death model, *DDX and DDL* exponential and logistic variants of the density-dependent speciation rate models, respectively, *yule2rate and yule3rate* multi-rate variants of the pureBirth model, *∆AIC*_*RC*_ the difference in AIC score between the best rate-constant (AIC_RC_) and rate-variable (AIC_RV_) models. The overall best-fit models were indicated with italic AIC value

### Biogeographic inferences

**Fig. 5:**
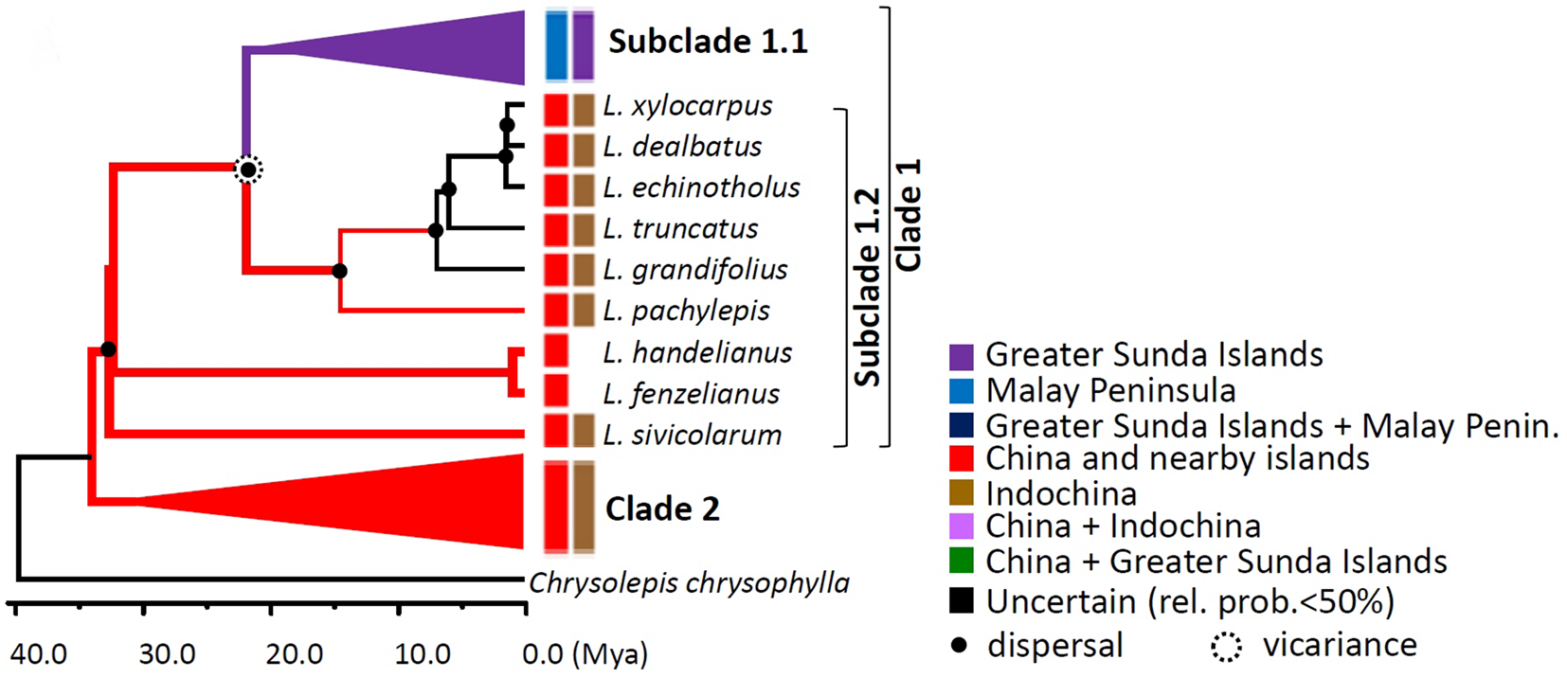
The species tree reconstructed by combined cpDNA *atp*B-*rbc*L spacer and nrITS data. Geographic distribution areas are displayed as colored boxes. Colored internodes indicate the ancestral geographic distribution areas reconstructed by DEC model. Lineages with bold and thin lines were derived from nodes (ancestral areas) with likelihood > 0.7 and > 0.5, respectively. Those with likelihoods < 0.5 are treated as unknown (black lineages). Dispersal and vicariance events inferred by S-DIVA are denoted

The ancestral area of the Asian stone oaks inferred from the DEC model is suggested to be continental Asia (or mainland China) by cpDNA + nrITS markers (Fig. 5 and Additional file 1: Figure S2A, -lnL = 76.94) in the early Oligocene period. The geographical distribution of extant *Lithocarpus* species is mostly a result of dispersal events and joined events of dispersal + vicariance, inferred by S-DIVA using combined cpDNA + nrITS markers (Fig. 5a, Additional file 1: Figure S2). Dispersal events are mostly inferred at clade 1, indicating that the species distributed in southern areas were mostly contributed by the Miocene southward expansions.

The southward colonization history of the common ancestor of these southern species from continental Asia could be traced back to 21.78 ~ 14.48 Mya (Figs. 2a and 5). This timing also matched the Miocene Climatic Optimum (MCO). Furthermore, the dispersal-related events followed by vicariance explained the far-distant close relatives. The S-DIVA inference from combined data showed a southward expansion followed by a vicariance that separated the Greater Sunda Islands from the areas of Indochina + continental Asia at 21.78 Mya (95% HPD: 9.92–25.28 Mya, posterior prob. = 0.4755) (Figs. 2a and 5).

### Temporal analyses of diversification rate shifts

**Fig. 6:**
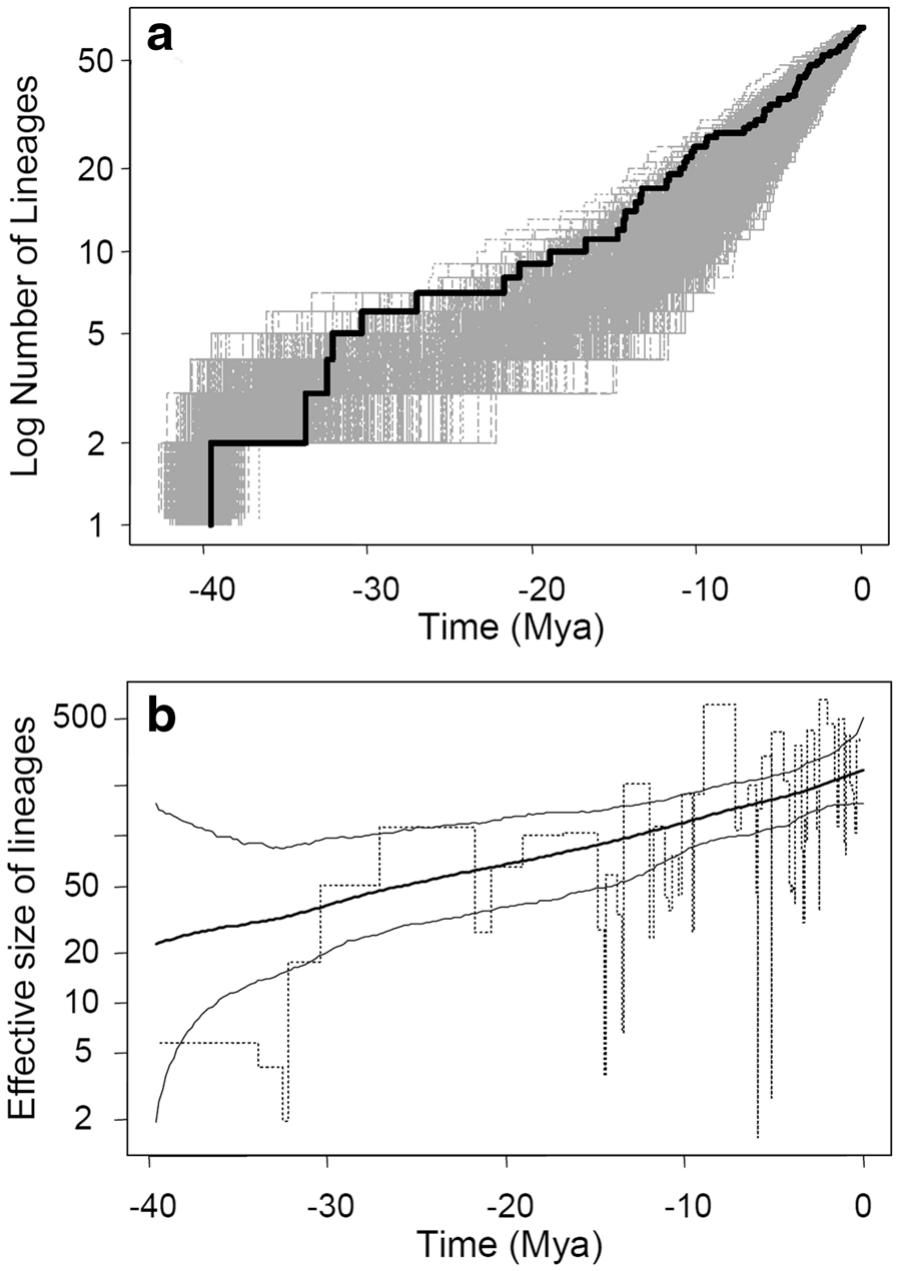
Skylines of the lineage-through-time (LTT) plots (**a**) and the effective size of lineages (**b**) of Asian *Lithocarpus* species from the cpDNA + nrITS tree. Grey regions of **a** indicate simulated LTT plots based on 1000 post-convergence Bayesian trees computed by BEAST. In **b**, the dashed lines represent the classic skyline plots, while the bold and thin solid lines indicate the skylines estimated with the rjMCMC and the corresponding 95% confidence intervals, respectively

In the temporal analyses, the LTT of the tree inferred from the combined markers cpDNA and nrITS (Fig. 6) showed two sharp increases in diversification rates at *c.* 35 Mya (the Early Oligocene) and 15 Mya (Middle Miocene) (see gray area of LTT plot simulated from 1000 post-convergence trees), which roughly correspond to the end of the Grande Coupure and the MCO, respectively.

### Diversification rate estimation considering incomplete sampling

The BAMM analysis that considered 20% sampling fractions revealed a temporal change rather than spatial heterogeneity in the diversification rate of *Lithocarpus*. The effective number of sampling sizes (ESS) for the log-likelihood and the rate-shift-event estimation are 2249.43 and 4217.825, verifying the credibility of the inference of simulation. From 4501 simulation samples, the shift posterior distribution of 0, 1, 2, and 3 times rate-shift events are 0.97, 0.032, 0.0011, and 0.00022, respectively, indicating that a no-diversification rate shift between clades is the best evolutionary scenario for the diversification rate shift. We further estimated the speciation rates of tropical lineages (the southern lineages: GSI + Malay Peninsula), subtropical lineages (the northern lineages: China + Indochina), and the whole tree (genus *Lithocarpus*). The rates were 0.551 (5%–95% quantile: 0.357–0.799), 0.540 (5%–95% quantile: 0.342–0.806), and 0.543 (5%–95% quantile: 0.348–0.803), respectively, revealing rate homogeneity among clades and a slight, congruent positive skewness of diversification rate (i.e., speciation rate > extinction rate, Fig. 6). The phylo-plot and density-plot of the net diversification rate revealed a congruent trend towards a slight increase of the net diversification rate among different clades, suggesting a homogeneous diversification rate in space and temporal change of diversification rates in whole *Lithocarpus* lineages (Fig. 5). These estimates suggested that although the species composition of *Lithocarpus* differed among localities, the speciation and net diversification rates were unrelated to geographical distribution.

The dynamics of effective lineage sizes estimated by rjMCMC sampling represent changes in genetic diversity over time (Opgen-Rhein et al. 2005), which revealed a constant rate of cladogenesis on the logarithmic axis by combined cpDNA and nrITS markers (Fig. 6b). The skylines of effective sizes of lineages inferred by the rjMCMC approach revealed more smooth curves of diversification rates, whereas the classic skylines revealed the intuitional estimates of lineage sizes at each time interval. Severe fluctuations of effective sizes of lineages at 15 ~ 13 Mya in the Middle Miocene followed by the Late Miocene explosion of lineages at *c.* 9 ~ 7 Mya and another fluctuation of lineage sizes at the Miocene-Pliocene boundary (6 ~ 5 Mya) revealed in the classic skyline plot suggest the conspicuous late-shifts of diversification rates in *Lithocarpus* (Fig. 6b).

### Multiple originations of the indigenous stone oaks on mainland China’s offshore islands

According to the species tree, the island species were paraphylies or polyphylies, suggesting that these species might have colonized the island multiple times and could have evolved independently. The twelve *Lithocarpus* species on the tropical island of Hainan are sparsely distributed in the phylogeny (Fig. 2a). Two of the three endemic species in Hainan (*L. handelianus* and *L. fenzelianus*) are sister-grouped with short TMRCA at *c.* 0.95 Mya (95% HPD: 0–17.35 Mya), but they are distinct from another endemic species *L. naiadarum* by a long coalescent history [about 33.91 Mya (95% HPD: 25.98–41.35 Mya)], suggesting at least two origins for the Hainan endemic species. The inference of multiple colonization events is also shown on the subtropical island of Taiwan. The Taiwanese species are phylogenetically located in one of the major clades but still sparsely distributed around the tree (Fig. 2a). The coalescent history of Taiwanese indigenous species could be traced back to 30.46 Mya (95% HPD: 9.74–27.74 Mya) and the TMRCA of any two species is longer than 5 Mya (Fig. 2a), which is older than the formation time of Taiwan Island [< 5 Mya, (Chi et al. 1981; Teng 1996; Wang et al. 2002)], indicating multiple colonization times instead of in situ radiation. The temperate island of Japan has only two species, *L. glaber* and *L. edulis*. The former species is also widely distributed in China and Taiwan, while the latter is endemic to Japan. These two Japanese species could have coalesced back to 3.82 Mya (95% HPD: 0.27–6.31 Mya), but the endemic one, *L. edulis*, is sister-grouped with another species, *L. brevicaudatus* (distributed in China and Taiwan, but not in Japan), with very short TMRCA at 0.18 Mya (95% HPD: 0–5.01 Mya) (Fig. 2a). The geological isolation could have differentiated ancestral populations of the common ancestor of *L. brevicaudatus* and *L. edulis* and resulted in the recent speciation of the island species *L. edulis*.

## Discussion

### Intricate patterns of phylogenetic relationships as inferred from chloroplast and nuclear markers

The different evolutionary trajectories for nuclear and plastid markers may lead to different phylogenetic inferences. The cpDNA tree (Fig. 3, Additional file 1: Figure S1A) and the nrITS tree (Fig. 4, Additional file 1: Figure S1B) share a similar pattern for the early-derived major groupings but are incongruent in the recently derived lineages, suggesting a major influence of biogeographic effects on *Lithocarpus* evolution and less possibly for independent evolutionary trajectories based on chloroplast and nuclear genomes. Conflicts between these two genomes were often attributed to different evolutionary rates and reflect different biogeographic events such as pollen flow and seed dispersal (Ennos 1994; Mccauley 1995; Petit et al. 2005).

The broad and overlapping confidence intervals in the recently derived clades (Figs. 3 and 4, Additional file 1: Figure S1) indicate uncertainty in phylogenetic inference among closely related taxa. Phylogenetic discrimination between closely related species or between populations may be difficult because of the slow rate of lineage sorting (Hollingsworth et al. 2011), which may result in incorrect phylogenetic inferences in the terminal branches of tree topology. Therefore, we will focus the discussion in terms of global diversification and biogeographic patterns instead of fine-scaled inferences.

Using DNA sequence-based phylogeny assessed from only two DNA fragments to infer the biogeographic pattern of *Lithocarpus* is a trade-off between sample size and phylogenetic resolution. We used a large number of samples resourced mostly from published data, but the availability of relatively few markers might result in a bias of phylogeny. The major concern for the use of limited markers is that most biogeographic analyses are tree-dependent, and the tree quality determines the accuracy of the biogeographic inferences. A biased phylogenetic hypothesis can lead to an erroneous biogeographic inference. Therefore, in this study, integrated biogeographic inferences were obtained by comparing the analyses from combined marker sets and two single loci of different genomes and the contrasting results from different trees are further discussed.

### Biogeographic inferences

The ancestral area of *Lithocarpus* inferred from the combined-marker tree is located in mainland China (Fig. 5, Additional file 1: Figures S2 and S4), implying a continental-Asian origin of the genus. Except for the genera *Lithocarpus* and *Trigonobalanus* (cf. Manos and Stanford 2001), most extant species of Fagaceae are distributed in temperate areas, suggesting a temperate origin of Fagaceae, which was also evidenced by the fossil distribution (Crepet and Nixon 1989). Given the apparent ecological niche conservatism throughout the evolutionary history of *Fagaceae*, it seems more likely that the subtropical and tropical genus *Lithocarpus* originated in a temperate region and expanded southward with adaptive divergence (Manos and Stanford 2001). The southward expansion was extended to the Greater Sunda Islands (Fig. 5, Additional file 1: Figures S2–S4). The estimated time of the southward migration places it in the Early Miocene, during which the sea level had not begun to rise and Sundaland was not yet submerged (Hall 2002), showing no or less relationship between the southward range expansion and recent dramatic climate changes, such as the Quaternary glacial oscillations. In fact, the early long-distance dispersal events did not occur only in Southeast Asia. Oligocene leaf fossils of the extant *Lithocarpus* analogs, *L. saxonicus*, discovered in Europe (East Germany) suggest a westward expansion of ancestors of extant *Lithocarpus* (Kvaček and Walther 1989). However, another example of out-of-Asia dispersal of *L. densiflora* into North America in the mid-Eocene (Manos and Stanford 2001) is paradoxical because this species is now recognized as a novel genus *Notholithocarpus* which is sister-grouped with *Quercus* instead of *Lithocarpus* (Manos et al. 2008). The Late-Miocene and Early Pliocene sea-level fluctuations that accompanied the disjunction and connection of Sunda Islands (Susilohadi et al. 2009) might have caused continual divergences and secondary contact with *Lithocarpus* species and resulted in rapid diversification. The following Pliocene fragmentation of island blocks (Susilohadi et al. 2009) further accelerated species divergence (e.g., Cyrtudru, Atkins et al. 2001; murine rodents, Gorog et al. 2004; crabs, Klaus et al. 2013).

The MCO provided suitable environmental conditions for broad spreading, and the following Middle Miocene climatic transition that caused the ice sheet expansion and formation of land bridges (Holbourn et al. 2005) might have facilitated southward colonization of these continental species into the Sundaland region. In addition to this southward colonization, we found at least two independent and synchronized northward returns from the Greater Sunda Islands into continental Asia at roughly 12 Mya, the Middle Miocene, by cpDNA (Fig. 3) and at least two serial northward returns at 15.21 Mya (95% HPD: 1.8–14.94 Mya) and 4.63 Mya (95% HPD: 0.72–10.58 Mya) by nrITS (Fig. 4) analysis.

The colonization date inference (21.78 ~ 14.48 Mya, Figs. 2a and 5a) of Southeast Asian stone oaks supports Cannon and Manos’s (2003) diversification hypothesis of Middle-Miocene vicariance between Indochina and Borneo. Manos and Stanford (2001) suggested that the most ancient haplotypes were distributed in northern Borneo and Indochina. The S-DIVA analysis with consideration of the scenario of vicariance rather than the DEC model implied serial dispersal events from the ancestral area of mainland China south towards Indochina and Borneo, indicating that the Borneo species were descended from the continental-Asian immigrants (Fig. 5). Our biogeographic inferences support Cannon and Manos’s (2003) hypothesis of Middle-Miocene vicariance between Indochina and Borneo, but the ancestral area of *Lithocarpus* is probably in mainland China, further north than Cannon and Manos’s (2003) inference of Borneo and Indochina. Furthermore, the Middle-Miocene long-distance dispersals also support the later in situ diversification inferred by Cannon and Manos (2003).

### Diversification rate shift of Asian stone oaks

The rejection of constant diversification rate models (Tables 1 and 2) and the gradual increase of certain diversification rate shifts (Fig. 6a and d) supported the rejection of the stochastic model of ecological drift at long-term time scales (Benedetti-Cecchi et al. 2008)—i.e., that the Asian stone oaks are a group of non-neutral species responsive to heterogeneous environments. The maternally inherited cpDNA that reflects seed-mediated biogeographic events revealed a more obvious diversification rate shift during the Miocene (*c.* 15 Mya, Fig. 6b and e) than the biparentally inherited nrITS marker (Fig. 6c and f). The Miocene southward colonization by long-distance seed dispersal plays an important role in the extant species diversity of Asian stone oaks (Fig. 2a and b; the blue arrows). The lineages of clade 1 experienced southward colonization and an increased diversification rate at the Middle-Miocene (ca. 15 Mya, Fig. 2b), while lineages of the continental Asian clade 2 revealed a later increase of diversification rate since the early Pliocene (*c.* 5 Mya, Fig. 2c). Ancient Sundaland did not separate during the Miocene but narrowed from the Middle Miocene (Hall 2002). Restricted routes for long-distance dispersal in addition to climatic deterioration might have obstructed the range expansion and accelerated population differentiation (or species divergence) in Borneo. In contrast, the topography of mainland China did not vary as did that of Sundaland (Hall 2002; Scotese 2014), so the diversification rate of clade 2 in Fig. 2 did not reveal a rate-transition in the Middle Miocene (Fig. 2c).

However, the single-marker analyses revealed a constant growth of diversification rate in subclades cp-1, cp-2 (Fig. 3), and ITS-I (Fig. 4), but showed a rate-decrease pattern during the Miocene in subclade ITS-II (*c.* 23 ~ 5 Mya, Fig. 4). The constant rate increase in subclades of cpDNA (Fig. 3), and the shift in the whole tree topology (Fig. 6b), were caused by different diversification rates of subclades cp-1 (higher rate increase) and cp-2 (lower rate increase) (Fig. 3). Before the Middle Miocene (ca. 15 Mya), the numbers of continental Asian species were higher than those in southern Sundalands, but the southward colonization of stone oaks and the more severe geotopological changes to Sundaland caused a faster rate of species accumulation in Sundaland (the Greater Sunda Islands) than in continental Asia. Clade ITS-II might reflect a counteraction of frequent interspecific pollen flow and restricted seed dispersal in continental Asia since the Middle Miocene. The early Middle Miocene had an optimal climate (i.e., the MCO) with subsequent Middle Miocene disruption, during which the mid-latitude arid belt replaced the humid climate in East Asia (Liu et al. 2009), which caused an expansion of grasslands and restriction of forested areas (Kurschner et al. 2008). This explains the seed-mediated differentiation. In addition, the abundant insects with rapid evolutionary rates during the Miocene (Nyman et al. 2012) could have created opportunities for successful interspecific pollen flow of entomophilic stone oaks.

In addition to the principal diversification rate shift in the Middle Miocene (*c.* 15 Mya, Fig. 2b), we assessed two minor rate shifts at the Miocene-Pliocene boundary (*c.* 5 Mya) and Pliocene–Pleistocene boundary (*c.* 2.5 Mya). The former minor diversification rate shift was caused by continental Asian lineages of subclade 2 (Fig. 2c), while the latter was caused by lineages of subclade 1 (Figs. 2b and 4b). The climate since the end of the MCO gradually became colder until drastic climatic changes occurred at the end of the Miocene (Hodell and Kennett 1986) that restricted forest expansion on the Asian landmass. Restricted habitats (refugia) obstructed the gene flow with other populations and increased population differentiation, which accelerated speciation rates of the continental stone oaks in Asia, although the risk of extinction was also increased. Climatic oscillations provided suitable corridors for species survival and dispersal (Nakamura et al. 2012) that may have facilitated species diversification by recurrent extinctions and abrupt speciation from open niches (Dynesius and Jansson 2000).

In contrast, the species distributed in relatively warm Sundaland were less affected until the Pliocene–Pleistocene boundary, during which Sundaland started to break up. The second minor diversification rate shift since the Pliocene–Pleistocene boundary (*c.* 2.5 Mya) reflects the drastic Quaternary climatic change and periodic glacial periods. Quaternary fluctuations in biota transitions were commonly adopted to explain the current biodiversity (Alexeeva and Erbajeva 2005; Graham et al. 2003). Climatic changes at the Quaternary that predominantly attended dispersal, isolation, and local extinction to foster speciation could explain the increased diversification rates (Gavrilets and Losos 2009; Graham et al. 2003).

### Multiple originations of insular stone oaks

After the southward colonization, the expansion and migration of *Lithocarpus* led to colonization of islands around continental Asia and Sundaland. Because of the distinct phylogenetic positions of island species in Japan, Taiwan, and Hainan, the hypothesis of multiple colonization events for each island is supported rather than in situ diversification. The deep branches of the cpDNA tree suggest long histories of seed colonization (Fig. 3), implying that the high species diversity of stone oaks in Taiwan and Hainan could be due to multiple origins instead of rapid speciation from single lineages (i.e., radiation). Furthermore, the formation time of Taiwan and Hainan was younger (since the Pliocene) than the formation of the landmass of Sundaland, i.e., Borneo (since at least the end of the Jurassic, Cracraft 1988), and the area of those two subtropical islands was also much more smaller than the landmass of Sundaland. These factors led to fewer species (or taxa) in Taiwan (16 spp.) and Hainan (24 spp.) than in Borneo (73 spp.) (Huang et al. 1999; Liao 1996; Soepadmo 1972). However, the endemism in Taiwan (8/16) was larger than in Borneo (23/73), other tropical islands (Hainan: 6/24, Philippines: 13/32, Sumatra: 4/33, Java: 4/17, Sulawesi: 0/4), and the Malay Peninsula (8/48) (Soepadmo 1972). Diversity of stone oaks in the Greater Sunda Islands was thought to be a consequence of prolonged in situ diversification (Cannon and Manos 2003) and is supported by our biogeographic analysis (Fig. 5, Additional file 1: Figures S2–S4), while the rapidity of speciation seems lower than the multiple-source colonization over a short span in Taiwan. The short distance between Taiwan and China (180 km on average) and the shallow continental shelf of the Taiwan Strait (100 m depth on average) joined to continental Asia repeatedly during glacial epochs, and led to multiple colonization between China and Taiwan, which resulted in periodic dispersal and isolation between species congeners (compared to Hainan Island: 18 km from China, 44 m depth in the Qiongzhou Strait, which reduced the isolation effect). The synergy of genetic isolation and adaptation to the rugged topography could have accelerated the speciation process (Bittkau and Comes 2005), which could be the cause of high endemism of Taiwanese stone oaks. In Taiwan, *Lithocarpus* species are highly diverged, with 14 species recognized and 7 species as endemic (*L. formosana*, *L. dodonaeifolius*, *L. shinsiensis*, *L. nantoensis*, *L. konishii*, *L. kawakamii,* and *L. lepidocarpus*) (Yang et al. 2014) usually in central and southern parts of the island, mixing with other species of Fagaceae, Lauraceae and coniferous forests at altitudes of 150–2700 m. Ecologically, seven species usually grow on wind-facing slopes in tropical or subtropical forests (Yang et al. 2014).

### Temporal change in diversification rate of Asian stone oaks

Both topological (Table 1) and temporal analyses (Fig. 4) of diversification rates suggest that *Lithocarpus* species were slowly accumulated and formed persistent lineages through time, supporting Thorne’s (1999) theory that East Asia serves as a living museum (refugium) to preserve archaic flora (cf. Favre et al. 2016). Such temporal increases in the synchronous diversification rate in subtropical and tropical lineages indicates that (1) the southward founder event did not accelerate the diversification rate and (2) the diversification rate shift of Asian stone oaks was spatially independent. Since the species composition and climate differ between the subtropics and tropics, the congruent patterns of diversification rates of stone oaks in these two areas suggest that the evolution of *Lithocarpus* may fit the stochastic model of ecological drift (Benedetti-Cecchi et al. 2008) rather than the ecological opportunity model (Yoder et al. 2010). The ecological opportunity theory posits an increased rate of morphological or species diversification due to a reduction in interspecific competitive pressures (i.e., ecological release) when novel habitats were colonized. In contrast, under the stochastic model, species assemblages in an environment are the consequence of random variation in the fundamental biological processes of birth, death, and migration (Bell 2000), in which environmental heterogeneity is not responsible for species diversity (Maurer and McGill 2004). One of the characteristics of the ecological opportunity theory is adaptive radiation (Losos and Mahler 2010; Yoder et al. 2010). Species that meet the ecological opportunity model have shorter life histories and are more sensitive to environmental changes—e.g., birds (Norman and Christidis 2016; Schweizer et al. 2014), fishes (Arbour and Lopez-Fernandez 2016; Silva et al. 2016), ferns (Sundue et al. 2015), and Gentianaceae (von Hagen and Kadereit 2003). Stone oaks with longer life histories could not instantaneously respond to environmental variations spatially, but in the long process of time, diversity could accumulate from different archaic sources (refugia). Such a “living museum” model of the diversification patterns of genus *Lithocarpus* could be explained by the hypothesis of multiple origins with in situ diversification of insular species.

## Conclusions

The diversification of Asian stone oaks is related to dispersal events in association with past climatic changes. The diversification rate heterogeneity across phylogenetic trees instead of punctuated rate shifts across clades signifies that the diversification events were related to global geohistory rather than episodic local events. Based on the coalescent species tree inferred by nuclear and chloroplast genome markers, we propose to revise the former hypothesis of a tropical origin to a subtropical origin and modify the vicariance hypothesis to one of dispersal with variance for explaining the in situ diversification of tropical stone oaks. The boundaries of geological times (i.e., the Oligocene–Miocene, Miocene-Pliocene, and Pliocene–Pleistocene) and the occurrence of drastic climate changes during the Middle-Miocene matched perfectly with the diversification rate shifts in *Lithocarpus*. Such drastic climatic changes at chronological times which led to restricted gene flow within continents might have accelerated species divergence and promoted diversification. The genus *Lithocarpus* serves as a good model for tropical-subtropical forest trees in reflecting environmental changes. Furthermore, our results suggest multiple origins of the newly originated indigenous species on subtropical continental islands, leading to high levels of insular biodiversity. An excess of high frequency endemics on Taiwan Island suggests the synergistic consequences of adaptive divergence and experienced secondary contacts between (isolated) specific congeners. However, we still need genomic data from a larger population to test the hypothesis of adaptive-divergence-driven in situ diversification.

## Additional file

**Additional file 1: Table S1.** Collecting sites and distribution. **Table S2.** Best substitution models for the *atp*B-*rbc*L and ITS used in the Bayesian analyses. **Table S3.** T Statistical summary of asymmetric values for the among-lineage diversification rate variation in the phylogenetic topologies. **Table S4.** Testing for diversification rate variation models by ∆AIC_RC_ test statistic. **Figure S1.** The phylogenetic tree reconstructed by cpDNA *atp*B-*rbc*L spacer and nrITS under the Yule’s pure-birth speciation model. Samples that were identified as the same species but represented at different clades in the gene trees (supplementary) were separated to different OTUs. Bold lines indicate the lineages grouping with posterior probability > 80%; node labels are the splitting time (unit: mya); node bar is the 95% highest posterior density interval (HPD) of the splitting time. The nodes probably have diversification rate shift were labeled by nodes *i* and *ii* of cpDNA tree and nodes *a* and *b* of ntITS tree. The testing results of diversification-rate shift of descendents of nodes *i*, *ii*, *a*, and *b* inferred by delta-statistics were indicated in the inner table. **Figure S2.** Biogeographic inferences by cpDNA+nrITS (Fig. 2A). (A) Biogeographic inference under the DEC model. Lineages with bold and thin lines indicate the lineages derived from nodes (ancestral areas) with likelihood > 0.7 and > 0.5, respectively. Likelihood less than 0.5 are treated as unknown (black lineages). (B) Biogeographic inference under the S-DIVA model. The dispersal (d) or vicariance (v) events with a posterior probability > 0.5 are marked in the node. The yellow dots indicate the events of range transition inferred by both dispersal and vicariance events. The open dots indicate that thebiogeographic distribuion of deriving lineages were explained as consequences of dispersal events. **Figure S3.** Biogeographic inferences by cpDNA (Fig. 3). (A) Biogeographic inference under the DEC model. (B) Biogeographic inference under the S-DIVA model. **Figure S4.** Biogeographic inferences by nrITS (Fig. 4). (A) Biogeographic inference under the DEC model. (B) Biogeographic inference under the S-DIVA model.

## Abbreviations

DEC: dispersal–extinction–cladogenesis
MCO: Miocene climatic optimum
CTAB: cetyl trimethylammonium bromide
PCR: polymerase chain reaction
nrITS: nuclear ribosomal internal transcribed spacer
LTT: lineage-through-time
rjMCMC: reversible jump Markov chain Monte Carlo
GTR: general-time-reversible substitution model
UCLN: uncorrelated lognormal
