# Supplementary material for: Nuclear and chloroplast DNA phylogeography suggests an Early Miocene southward expansion of Lithocarpus (Fagaceae) on the Asian continent and islands
Source: Bot Stud. 2018 Nov 8;59:27. doi: 10.1186/s40529-018-0244-8 (PMC6223401; doi:10.1186/s40529-018-0244-8)
Supplement: Supplementary file 1 — Additional file 1: Table S1. Collecting sites and distribution. Table S2. Best substitution models for the atpB-rbcL and ITS used in the Bayesian analyses. Table S3. T Statistical summary of asymmetric values for the among-lineage diversification rate variation in the phylogenetic topologies. Table S4. Testing for diversification rate variation models by ∆AICRC test statistic. Figure S1. The phylogenetic tree reconstructed by cpDNA atpB-rbcL spacer and nrITS under the Yule’s pure-birth speciation model. Samples that were identified as the same species but represented at different clades in the gene trees (supplementary) were separated to different OTUs. Bold lines indicate the lineages grouping with posterior probability > 80%; node labels are the splitting time (unit: mya); node bar is the 95% highest posterior density interval (HPD) of the splitting time. The nodes probably have diversification rate shift were labeled by nodes i and ii of cpDNA tree and nodes a and b of ntITS tree. The testing results of diversification-rate shift of descendents of nodes i, ii, a, and b inferred by delta-statistics were indicated in the inner table. Figure S2. Biogeographic inferences by cpDNA+nrITS (Fig. 2A). (A) Biogeographic inference under the DEC model. Lineages with bold and thin lines indicate the lineages derived from nodes (ancestral areas) with likelihood > 0.7 and > 0.5, respectively. Likelihood less than 0.5 are treated as unknown (black lineages). (B) Biogeographic inference under the S-DIVA model. The dispersal (d) or vicariance (v) events with a posterior probability > 0.5 are marked in the node. The yellow dots indicate the events of range transition inferred by both dispersal and vicariance events. The open dots indicate that thebiogeographic distribuion of deriving lineages were explained as consequences of dispersal events. Figure S3. Biogeographic inferences by cpDNA (Fig. 3). (A) Biogeographic inference under the DEC model. (B) Biogeographic inference under th [file 40529_2018_244_MOESM1_ESM.docx]

**Nuclear and chloroplast DNA phylogeography suggests the Early Miocene southward expansion and Middle-Late Miocene northward returns of *Lithocarpus* (Fagaceae) in Asian continent and islands**

Chih-Kai Yang^1,2^, Yu-Chung Chiang ^3^, Bing-Hong Huang ^1^, Li-Ping Ju ^4^, , Pei-Chun Liao ^1^*

^1^ Department of Life Science, National Taiwan Normal University, 88, Ting-Chow Rd, Sec 4, Taipei 116, Taiwan.

^2^ The Experimental Forest, College of Bio-Resources and Agriculture, National Taiwan University, 12, Sec. 1, Chien-Shan Rd., Nantou 55750, Taiwan.

^3^ Department of Biological Sciences, National Sun Yat-sen University, 70, Lien-Hai Rd., Kaohsiung 80424, Taiwan.

^4^ Botanical Garden Division, Taiwan Forestry Research Institute, 53, Nan-Hai Rd., Taipei 10066, Taiwan.

**Additional Data**

**Table S1 ~ S4**

**Figures S1 ~ S4**

Table S1 Collecting sites and distribution

| Species | Collecting site | Distribution area | cpDNA | nrITS | Voucher |
| --- | --- | --- | --- | --- | --- |
| *Chrysolepis chrysophylla* | NCBI | USA | FJ185062 | AF389087 |  |
| *C. sempervirens* | NCBI | USA |  | AY040369 |  |
| *Lithocarpus amygdalifolius* | Fushan, Guangxi | Taiwan, China, Hainan, Vietnam | KF992718* | KJ685163*  KJ685164* | 031844  5353 |
| *L. balansae* | NCBI | China, Vietnam, Laos |  | EU195795 |  |
| *L. bancanus* | NCBI | Sumatra, Malay Peninsula, Borneo | AB124945 |  |  |
| *L. beccarianus* | NCBI | Borneo | AY182835  AY182836  AY182837 | AF389101 |  |
| *L. bennettii* | NCBI | Sumatra, Malay Peninsula, Borneo | AY182838 | AF389093  AY040412 |  |
| *L. blumeanus* | NCBI | Borneo | AY182839 | AF389091 |  |
| *L. brevicaudatus* | Fushan | Taiwan, China, Hainan | KF992719*  KF992720* | KJ685165* | Ju2055 |
| *L. bullatus* | NCBI | Borneo | AY182840 | AY040409 |  |
| *L. cantleyanus* | NCBI | Malay Peninsula, Borneo | AY182841 |  |  |
| *L. calophyllus* | Nanling | China | KF992724*  KF992725*  KF992726*  KF992727*  KF992728* | KJ685166* | Ju2367-1~5 |
| *L. chrysocomus* | Nanling | China | KF992721* | KJ685167* | Ju2413 |
| *L. cleistocarpus* | Emei | China | KF992722*  KF992723* | KJ685168* | Ju2029  Ju2106 |
| *L. clementianus* | NCBI | Malay Peninsula, Borneo | AY182842  AY182843  AY182844  AY182845  AY182846 | AF389107 |  |
| *L. conocarpus* | NCBI | Sumatra, Malay Peninsula, Borneo, Java | AY182847  AY182848  AY182849  AY182850  AY182851 | AF389095  AY040417 |  |
| *L. cooperatus* | NCBI | Malay Peninsula, Borneo, Philippines | AY182852 | AY040406  AY040407 |  |
| *L. cornea* | NCBI | China, Taiwan, Vietnam | KF992729* | KJ685169*  AY040440 | 1676 |
| *L. cyrtocarpus* | NCBI | China, Vietnam | AY182853 |  |  |
| *L. dasystachyus* | NCBI | Borneo | AY182854  AY182855 |  |  |
| *L. dealbatus* | NCBI | China, Vietnam, Bhutan, India, Laos, Myanmar, Thailand, | AY182856 | AY040430 |  |
| *L. dodoniifolia* | Fushan | Taiwan | KF992730* | KJ685170* | 772-1 |
| *L. echinifer* | NCBI | Borneo | AY182858 | AF389089  AY040399 |  |
| *L. echinophorus* | NCBI | China, Laos, Myanmar, Vietnam | AY182859 | AY040437 |  |
| *L. echinotholus* | NCBI | China, Vietnam | AY182860 | AY040424 |  |
| *L. edulis* | NCBI | Japan | AB124946  AY182861 | AY040439 |  |
| *L. elegans* | NCBI | India, Pakistan, Burma, Sumantra, Malay Peninsula, Java, Borneo, Celebes | AY182862  AY182863 |  |  |
| *L. encleisocarpus* | NCBI | Peninsular Siam, Malay Peninsula, Sumantra, Borneo | AY182864  AY182865  AY182866  AY182867 | AF389094 |  |
| *L. ewyckii* | NCBI | Sumatra, Malay Peninsula, Borneo | AY182868  AY182870 | AY040415  AY040413 |  |
| *L. fenestratus* | NCBI | China, Bhutan, India, Laos, Myanmar, Sikkim, Thiland, Vietnam | AY182871  AY182872 | AY040443  AY040444  AY040449 |  |
| *L. fenzelianus* | Hainan | Hainan | KF992731* | KJ685171*  KJ685172* |  |
| *L. ferrugineus* | NCBI | Borneo | AY182874  AY182875 | AY040411  AY040414 |  |
| *L. formosana* | Fushan | Taiwan | KF992732* | KJ685173* | 952-7 |
| *L. glaber* | NCBI, Fushan | Taiwan, China, Japan | AB124947 | AY040435 | Ju2417 |
|  |  |  | KF992733*  KF992734*  KF992735*  KF992736*  KF992737* | KJ685174*  KJ685175*  KJ685176*  KJ685177*  KJ685178*  KJ685179* | Ju2440  Ju2441  Ju2472 |
| *L. gracilis* | NCBI | Sumatra, Malay Peninsula, Borneo | AY182877  AY182878 |  |  |
| *L. grandifolius* | NCBI | China, Bhutan, India, Laos, Nepal, Myanmar, Thailand | AY182879 | AY040436  AY040450 |  |
| *L. hancei* | NCBI, Fushan, Guangxi, Nanling | Taiwan, China, Hainan | KF992738*  KF992739*  KF992740*  KF992741*  KF992742*  KF992743*  KF992744* | AY040448  AY040451  KJ685180*  KJ685181*  KJ685182*  KJ685183*  KJ685184* | Ju2365  Ju2412  020418  91T  22-02  1552 |
| *L. handelianus* | Hainan | Hainan | KF992745* | KJ685185* |  |
| *L. harlandii* | Fushan, Guangxi, Nanling, Mangdang shan, Emei | Taiwan, China | KF992746*  KF992747*  KF992748*  KF992749*  KF992750*  KF992751*  KF992752*  KF992753*  KF992754*  KF992755* KF992756*  KF992757*  KF992758*  KF992759*  KF992760*  KF992761* | KJ685186*  KJ685187*  KJ685188*  KJ685189* | Ju2366  Ju2418  Ju2419  Ju2470  129-1~12 |
| *L. hatusimae* | NCBI | Philippines, Borneo | AY182880  AY182881  AY182882 | AY040410 |  |
| *L. havilandii* | NCBI | Borneo, Celebes | AY182883  AY182884  AY182885  AY182886  AY182887 | AF389092  AY040404  AY040405 |  |
| *L. henryi* | NCBI | China |  | EF057110 |  |
| *L. jacobsii* | NCBI | Malay Peninisula, Borneo | AY182898 |  |  |
| *L. kalkmanii* | NCBI | Borneo | AY182899  AY182900  AY182901  AY182902 | AF389102 |  |
| *L. kawakamii* | Fushan | Taiwan | KF992762*  KF992763* | KJ685190* | 2707  3938 |
| *L. keningauensis* | NCBI | Borneo | AY182903 | AF389106 |  |
| *L. konishii* | Fushan | Taiwan, Hainan | KF992764* | KJ685191*  KJ685192* | 1480  7-20 |
| *L. lampadarius* | NCBI | Malay Peninsula Borneo | AY182904  AY182905  AY182906 | AF389099  AY040433 |  |
| *L. laoticus* | NCBI | China, Vietnam, Laos |  | EU195797 |  |
| *L. lepidocarpus* | Fushan | Taiwan | KF992765* | KJ685193*  KJ685194* | 3200 |
| *L. leptogyne* | NCBI | Malay Peninsula, Borneo |  | AY040416 |  |
| *L. lindleyanus* | NCBI | Vietnam | AY182907 |  |  |
| *L. litseifolius* | Guangxi | China, Hainan, Laos, Myanmar, Vietnam | KF992766*  KF992767* | EF057112  KJ685195* | 970642 |
| *L. lucidus* | NCBI | Sumatra, Malay Peninsula, Borneo | AB124948  AY182908 | AF389088  AY040408 |  |
| *L. luteus* | NCBI | Borneo | AY182909  AY182910 | AF389096 |  |
| *L. meijerii* | NCBI | Borneo | AY182911 |  |  |
| *L. naiadarum* | Hainan | Hainan | KF992772*  KF992773* | KJ685196* |  |
| *L. nantoensis* | Fushan | Taiwan | KF992774* | KJ685197* | 950-4 |
| *L. nieuwenhuisii* | NCBI | Borneo Phillipine | AY182912 | AY040400 |  |
| *L. oleaefolius* | Nanling | China | KF992775* | KJ685198* |  |
| *L. pachylepis* | NCBI | China, Vietnam | FJ185065 | AY040441 |  |
| *L. pachyphyllus* | NCBI | China, Bhutan, India, Myanmar, Nepal, Sikkim | AY182913  AY182914 | AY040446  AY040447 |  |
| *L. palungensis* | NCBI | Borneo | AY182915 | AF389103  AY040420 |  |
| *L. paniculatus* | Nanling | China | KF992776*  KF992777* | KJ685199* | Ju2415  Ju2416 |
| *L. papillifer* | NCBI | Borneo | AY182916  AY182917  AY182918  AY182919 | AY040418 |  |
| *L. porcatus* | NCBI | Borneo | AY182920  AY182921  AY182922 |  |  |
| *L. pseudokunstleri* | NCBI | Borneo | AY182923 |  |  |
| *L. pulcher* | NCBI | Borneo | AY182924  AY182925  AY182926  AY182927 | AF389104  AY040421  AY040422  AY040423 |  |
| *L. revolutus* | NCBI | Borneo |  | AF389098  AY040434 |  |
| *L. taitoensis* | Fushan, Guangxi | Taiwan | KF992778*  KF992791* | KJ685200* | 1118  4259 |
| *L. rassa* | NCBI | Sumatra, Malay Peninsula, Borneo | AY182928 |  |  |
| *L. revolutus* | NCBI | Borneo | AY182929  AY182930 |  |  |
| *L. rosthornis* | Chinfo shan | China | KF992779*  KF992780*  KF992781* | KJ685201* | Ju2029  Ju2096  Ju2090 |
| *L. rotundatus* | NCBI | Java, Phillipine, Borneo | AY182931 | AF389090 |  |
| *L. rufovillosus* | NCBI | New Guinea |  | DQ499087 |  |
| *L. ruminatus* | NCBI | Borneo | AY182932  AY182933  AY182934  AY182936  AY182937  AY182938 | AF389097  AY040401  AY040403 |  |
| *L. sericobalanus* | NCBI | Borneo | AY182939  AY182940  AY182941  AY182942 | AF389105  AY040419 |  |
| *L. shinsuiensis* | Fushan | Taiwan | KF992782* | KJ685202* | 3622 |
| *L. sivicolarum* | Hainan | Chian, Hainan, Vietnam | KF992783* | KJ685203*  KJ685204*  KJ685205* |  |
| *L. skanianus* | Mangdang shan | China, Hainan | KF992784*  KF992785*  KF992786*  KF992787*  KF992788*  KF992789*  KF992790* | KJ685206*  KJ685207* | Ju2473-1~7 |
| *L. truncatus* | NCBI | China, India, Myanmar, Thailand, Vietnam | AY182944  AY182945  AY182946  AY182947 | AY040425  AY040428  AY040429  AY040431 |  |
| *L. turbinatus* | NCBI | Borneo | AY182948 | AF389100  AY040398 |  |
| *L. uvarifolius* | Mangdang shan | China | KF992792*  KF992793*  KF992794*  KF992795*  KF992796* | KJ685209*  KJ685210*  KJ685211*  KJ685212* | Ju2442-1~5 |
| *L. variolosus* | NCBI | China, Vietnam | AY182949 |  |  |
| *L. wallichianus* | NCBI | Peninsular Siam, Sumatra, Malay Peninsula | AB124949 |  |  |
| *L. xylocarpus* | NCBI | China, India, Laos, Myanmar, Vietnam | AY182950  FJ185066 | AY040426 |  |

* Sequences obtained in this study.

Emei: Emei Biological Resource Experimental Station, Sichuan 614206, China

Guizhou: Guizhou Botanical Garden, Chinese Academy of Sciences, Guiyang 550001, China

Fushan: Fushan Botanical Garden, Yilan 264, Taiwan.

Table S2 Best substitution models for the *atp*B-*rbc*L and ITS used in the Bayesian analyses

| Locus | Model | Parameters | BIC | AICc | *lnL* | (+*G*)^b^ | *R*^c^ |
| --- | --- | --- | --- | --- | --- | --- | --- |
| *atp*B-*rbc*L | GTR+G | 400 | 9062.484 | 5255.211 | -2226.016 | 0.47 | 1.22 |
| ITS | K2P+G | 253 | 14106.016 | 11875.352 | -5683.394 | 1.19 | 4.65 |

^a^Proportion of the invariable sites;

^b^Modeling of discrete gamma distribution;

^c^Transition/transversion bias;

K2P: Kimura 2-parameter model

GTR: general time reversible model

Table S3 T Statistical summary of asymmetric values for the among-lineage diversification rate variation in the phylogenetic topologies

|  | *I_C_* | *M_Π_** | *M_Π_* | *M_Σ_** | *M_Σ_* |
| --- | --- | --- | --- | --- | --- |
| cpDNA+ nrITS (Figure 2A) |  |  |  |  |  |
| Observed | 251 | -0.5855 | 1.50E-10 | 0.6747 | 50.2261 |
| Min ERM | 507 | -0.9566 | 1.93E-17 | 0.5302 | 44.0535 |
| Max ERM | 64 | -0.0856 | 0.0472 | 0.9256 | 61.3567 |
| 0.025 frequentile RR | 368 | -0.8315 | 1.08E-13 | 0.5918 | 47.8035 |
| 0.975 frequentile RR | 233 | -0.5207 | 4.01E-08 | 0.7284 | 54.2252 |
| --> TailPr | 0.0006 | 0.0003 | 3.00E-05 | 0.0001 | 6.00E-05 |
| cpDNA (Figure 3A) |  |  |  |  |  |
| Observed | 324 | -0.5129 | 4.33E-10 | 0.7196 | 66.2184 |
| Min ERM | 651 | -0.9533 | 5.95E-19 | 0.5418 | 55.7200 |
| Max ERM | 104 | -0.1478 | 0.0010 | 0.8833 | 73.5864 |
| 0.025 frequentile RR | 440 | -0.7518 | 1.98E-15 | 0.6262 | 60.3685 |
| 0.975 frequentile RR | 227 | -0.3930 | 2.91E-08 | 0.7900 | 68.394 |
| --> TailPr | 0.0051 | 0.0005 | 8.00E-05 | 0.0002 | 0.0002 |
| nrITS (Figure 4A) |  |  |  |  |  |
| Observed | 294 | -0.5483 | 1.43E-10 | 0.7035 | 58.2993 |
| Min ERM | 589 | -1.0168 | 2.51E-19 | 0.5244 | 50.1407 |
| Max ERM | 79 | -0.1281 | 0.0020 | 0.8990 | 67.3080 |
| 0.025 frequentile RR | 386 | -0.7540 | 3.50E-14 | 0.6198 | 55.2110 |
| 0.975 frequentile RR | 215 | -0.4135 | 9.37E-08 | 0.7744 | 62.4962 |
| --> TailPr | 0.0044 | 0.0023 | 0.0037 | 0.0059 | 0.0048 |
| cpDNA (Figure S1A) |  |  |  |  |  |
| Observed | 599 | -0.5573 | 3.72E-19 | 0.6878 | 103.0830 |
| Min ERM | 1112 | -0.8805 | 1.77E-28 | 0.5576 | 94.1847 |
| Max ERM | 220 | -0.2034 | 2.54E-07 | 0.8536 | 117.8260 |
| 0.025 frequentile RR | 854 | -0.7510 | 2.40E-23 | 0.6348 | 100.578 |
| 0.975 frequentile RR | 463 | -0.4276 | 8.03E-14 | 0.7606 | 110.5650 |
| --> TailPr | 0.0007 | 3.00.E-05 | 0.0001 | 0.0007 | 0.0022 |
| nrITS (Figure S1B) |  |  |  |  |  |
| Observed | 724 | -1.0221 | 2.02E-21 | 0.5400 | 60.0154 |
| Min ERM | 754 | -1.0083 | 5.14E-20 | 0.5637 | 60.8534 |
| Max ERM | 120 | -0.1426 | 0.0004 | 0.8893 | 80.0882 |
| 0.025 frequentile RR | 652 | -0.9238 | 9.54E-20 | 0.5688 | 62.8712 |
| 0.975 frequentile RR | 351 | -0.5370 | 1.53E-12 | 0.6977 | 70.1858 |
| --> TailPr | 0.0021 | 0.0005 | 0.0019 | 0.0017 | 0.0144 |

*I_C_* is the Colless’s tree imbalance index; *M_Π_* and *M_Σ_* are the nodal probability product and nodal probability sum of the tree, respectively; *M_Π_** and *M_Σ_** are modified versions of *M_Π_* and *M_Σ_* obtained through differential weighting of the individual equal-rate Markov nodal probabilities according to their species diversity. These indices display the diversification rate variation of the whole tree.

Table S4 Testing for diversification rate variation models by ∆AIC_RC_ test statistic

| Model | Log-likelihood | AIC | Parameter |
| --- | --- | --- | --- |
| cpDNA+ nrITS (Figure 2A) |  |  |  |
| pureBirth | -2.3419 | **6.684** | r1=0.101 |
| bd | -1.682121 | 7.364 | r1=0.076, a=0.405 |
| DDX | -1.972169 | 7.944 | r1=0.065, x=-0.136 |
| DDL | -2.342 | 8.684 | r1=0.101, k=1168951 |
| yule2rate | -1.083 | 8.166 | r1=0.0687, r2=0.112, st1=14.894 |
| yule3rate | 1.871 | **6.258** | r1=0.087, r2=9.936, r3=0.115, st1=5.939, st2=5.936 |
| ∆AIC_RC_ |  | 0.426 |  |
| cpDNA (Figure 3A) |  |  |  |
| pureBirth | 25.97718 | **-49.95435** | r1=0.120 |
| bd | 26.15159 | -48.30319 | r1=0.105, a=0.220 |
| DDX | 26.19223 | -48.38446 | r1=0.091, x=-0.082 |
| DDL | 25.97713 | -47.95426 | r1=0.120, k=1428290 |
| yule2rate | 27.806 | -49.612 | r1=0.063, r2=0.130, st1=15.636 |
| yule3rate | 31.18926 | **-52.37852** | r1=0.063, r2=0.665, r3=0.124, st1=15.636, st2=15.017 |
| ∆AIC_RC_ |  | 2.424 |  |
| nrITS (Figure 4A) |  |  |  |
| pureBirth | -5.870249 | 13.7405 | r1=0.086 |
| bd | -4.303511 | **12.60702** | r1=0.058, a=0.532 |
| DDX | -5.695618 | 15.39124 | r1=0.061, x=-0.105 |
| DDL | -5.870443 | 15.74089 | r1=0.086, k=1284297 |
| yule2rate | -2.426486 | 10.85297 | r1=0.073, r2=0.148, st1=2.380 |
| yule3rate | 1.189585 | **7.62083** | r1=0.075, r2=0.471, r3=0.112, st1=1.713, st2=1.457 |
| ∆AIC_RC_ |  | 4.986 |  |
| cpDNA (Figure S1A) |  |  |  |
| pureBirth | 103.309 | -204.617 | r1=0.121 |
| bd | 107.116 | **-210.231** | r1=0.076, a=0.574 |
| DDX | 106.169 | -208.338 | r1=0.046, x=-0.251 |
| DDL | 103.308 | -202.616 | r1=0.121, k=2195621 |
| yule2rate | 108.500 | -211.000 | r1=0.084, r2=0.153, st1=7.286 |
| yule3rate | 112.757 | **-215.514** | r1=0.095, r2=0.184, r3=0.024, st1=4.059, st2=0.328 |
| ∆AIC_RC_ |  | 5.283 |  |
| nrITS (Figure S1B) |  |  |  |
| pureBirth | 27.403 | -52.806 | r1=0.110 |
| bd | 31.284 | **-58.568** | r1=0.060, a=0.667 |
| DDX | 29.731 | -55.463 | r1=0.038, x=-0.306 |
| DDL | 21.496 | -38.991 | r1=0.140, k=174 |
| yule2rate | 31.106 | -56.213 | r1=0.103, r2=0.368, st1=0.227 |
| yule3rate | 34.049 | **-58.096** | r1=0.093, r2=9.384, r3=0.172, st1=2.055, st2=2.053 |
| ∆AIC_RC_ |  | -0.471 |  |

pureBirth, pure birth (Yule) model; bd, rate-constant birth-death model; DDX and DDL, exponential and logistic variants of the density-dependent speciation rate models, respectively; yule2rate and yule3rate, multi-rate variants of the pureBirth model; ∆AIC_RC_, the difference in AIC score between the best rate-constant (AIC_RC_) and rate-variable (AIC_RV_) models. r1, r2, r3, net diversification rates at stages 1, 2, and 3; st1 and st2, the first and the second rate-shift times; a, the extinction fraction extinction rate/speciation rate; x, the x parameter in the density-dependent exponetial model; k, the K parameter in the logistic density dependent model.


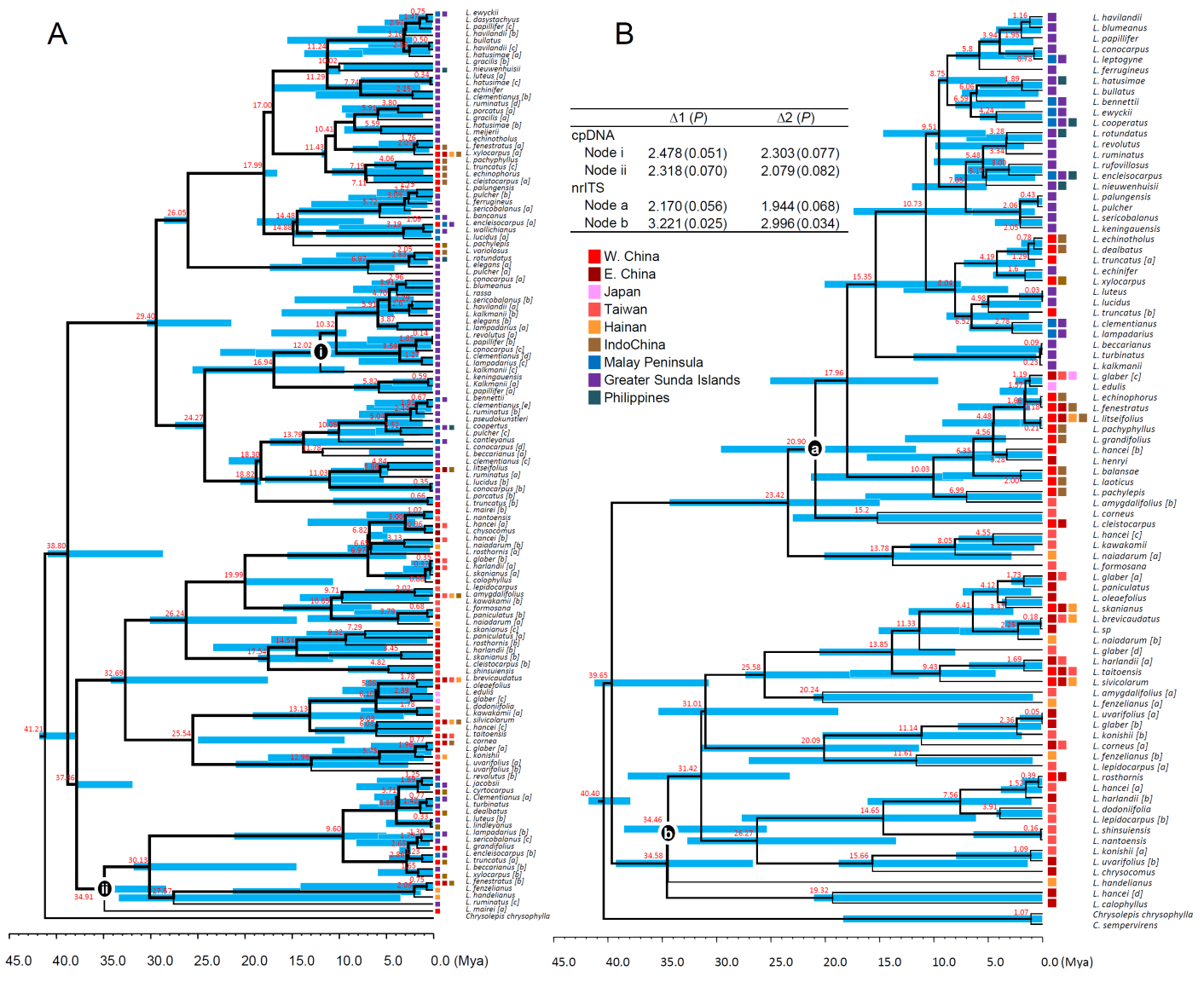


Figure S1 The phylogenetic tree reconstructed by cpDNA *atp*B-*rbc*L spacer and nrITS under the Yule’s pure-birth speciation model. Samples that were identified as the same species but represented at different clades in the gene trees (supplementary) were separated to different OTUs. Bold lines indicate the lineages grouping with posterior probability > 80%; node labels are the splitting time (unit: mya); node bar is the 95% highest posterior density interval (HPD) of the splitting time. The nodes probably have diversification rate shift were labeled by nodes *i* and *ii* of cpDNA tree and nodes *a* and *b* of ntITS tree. The testing results of diversification-rate shift of descendents of nodes *i*, *ii*, *a*, and *b* inferred by delta-statistics were indicated in the inner table.


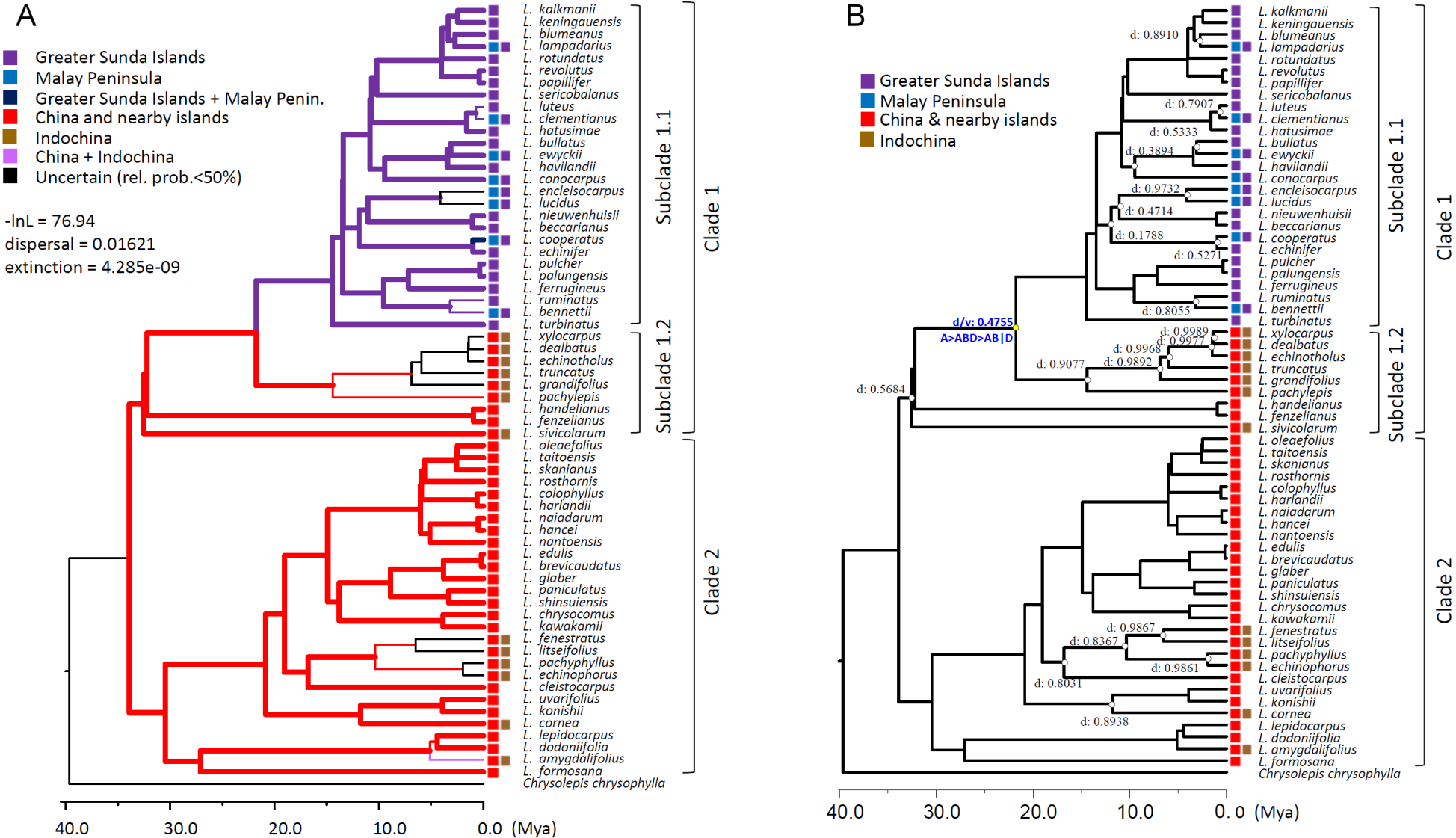


Figure S2 Biogeographic inferences by cpDNA+nrITS (Fig. 2A). (A) Biogeographic inference under the DEC model. Lineages with bold and thin lines indicate the lineages derived from nodes (ancestral areas) with likelihood > 0.7 and > 0.5, respectively. Likelihood less than 0.5 are treated as unknown (black lineages). (B) Biogeographic inference under the S-DIVA model. The dispersal (d) or vicariance (v) events with a posterior probability > 0.5 are marked in the node. The yellow dots indicate the events of range transition inferred by both dispersal and vicariance events. The open dots indicate that thebiogeographic distribuion of deriving lineages were explained as consequences of dispersal events.


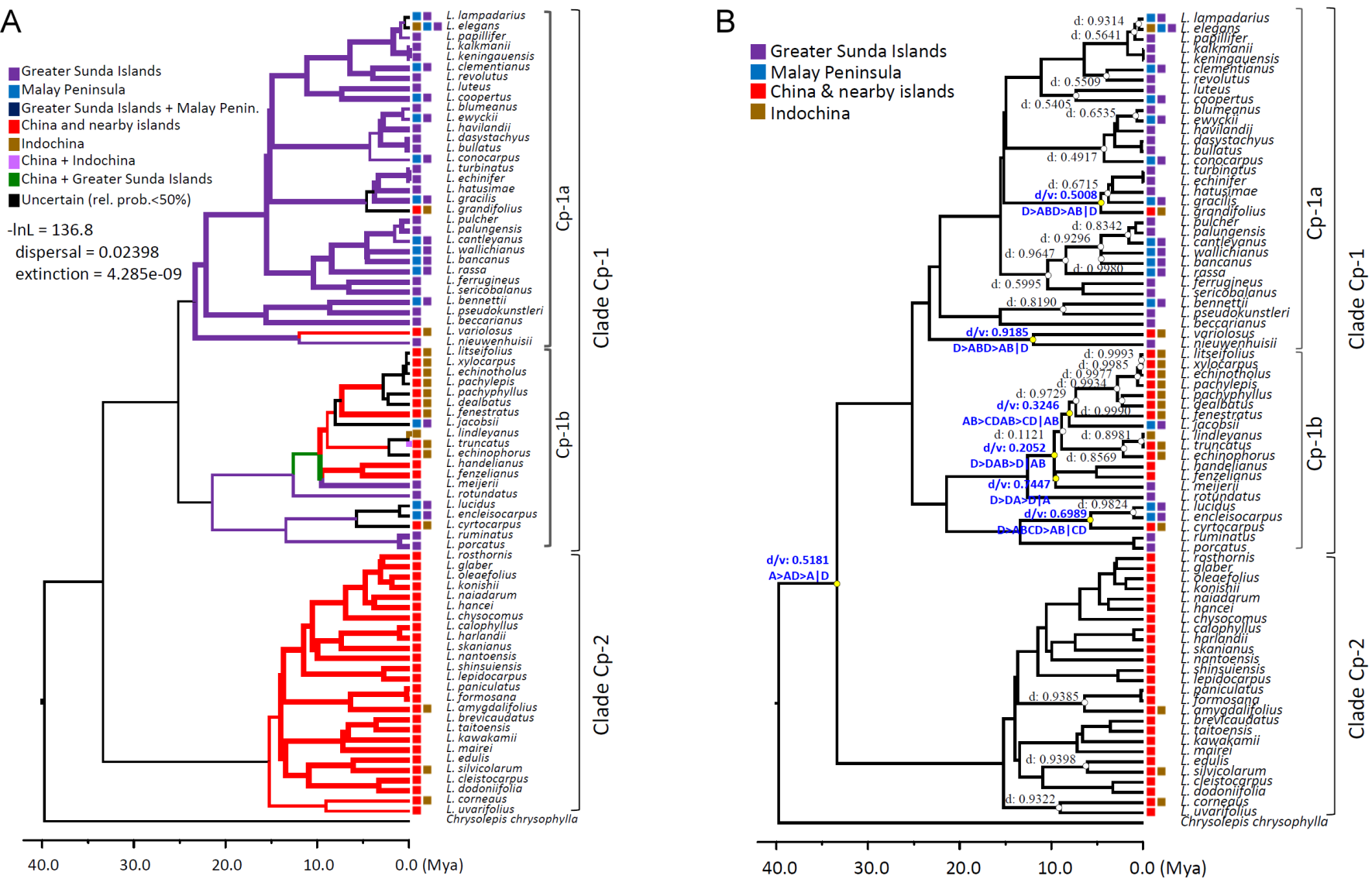


Figure S3 Biogeographic inferences by cpDNA (Fig. 3A). (A) Biogeographic inference under the DEC model. (B) Biogeographic inference under the S-DIVA model.


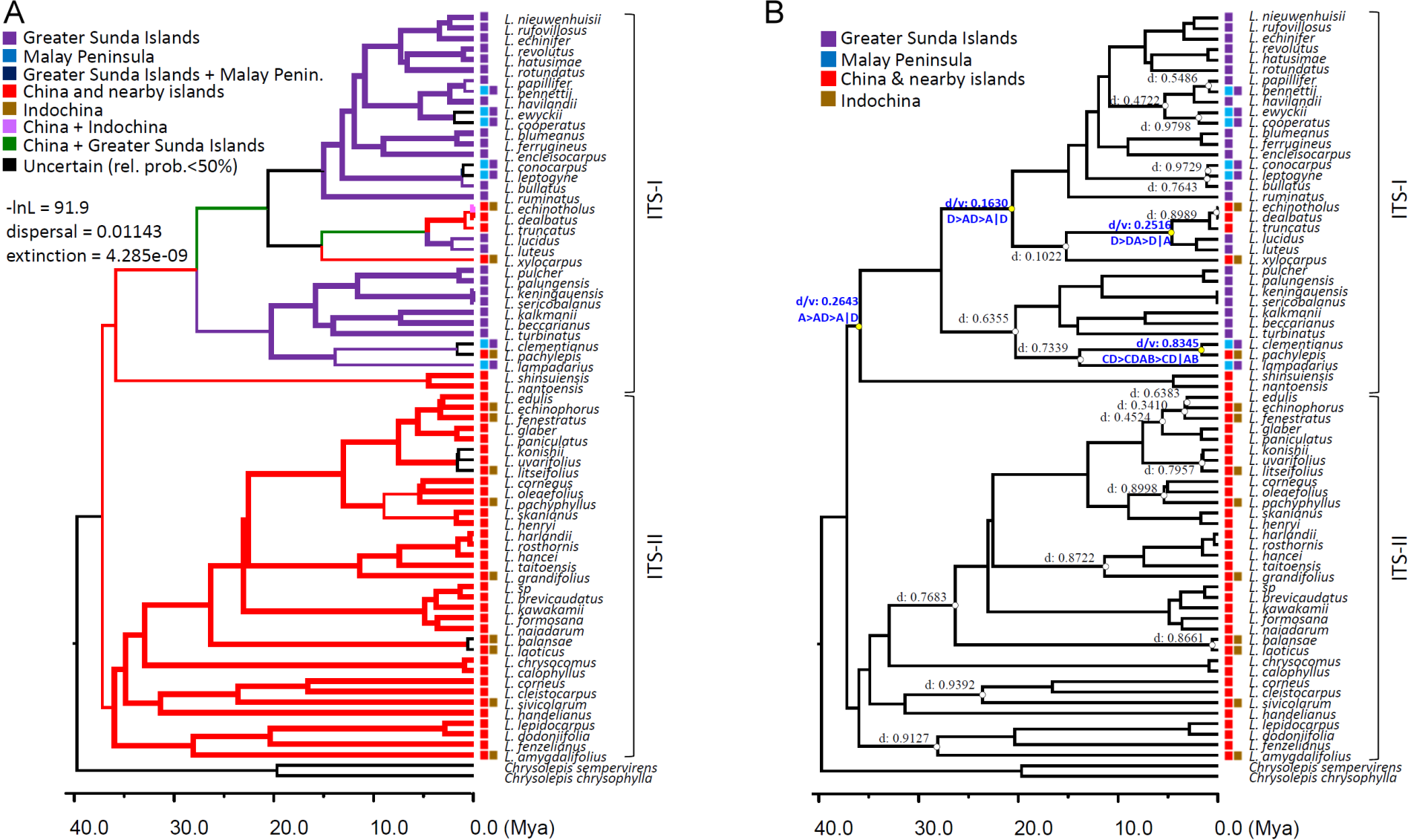


Figure S4 Biogeographic inferences by nrITS (Fig. 4A). (A) Biogeographic inference under the DEC model. (B) Biogeographic inference under the S-DIVA model.
